# Interventions for Childhood Central Obesity: A Systematic Review and Meta-Analysis

**DOI:** 10.1001/jamanetworkopen.2025.4331

**Published:** 2025-04-11

**Authors:** Setognal B. Aychiluhm, Utpal K. Mondal, Vivian Isaac, Allen G. Ross, Kedir Y. Ahmed

**Affiliations:** 1 Rural Health Research Institute, Charles Sturt University, Orange, New South Wales, Australia; 2 Institute of Public Health, College of Medicine and Health Sciences, University of Gondar, Gondar, Ethiopia; 3 School of Allied Health, Exercise & Sports Sciences, Faculty of Science & Health, Charles Sturt University, Albury, New South Wales, Australia; 4 Translational Health Research Institute, Western Sydney University, Campbelltown Campus, New South Wales, Australia

## Abstract

**Importance:**

The rapid rise in childhood central obesity and its cardiometabolic complications in adulthood highlight the urgent need for targeted global interventions.

**Objective:**

To examine the association of lifestyle, behavioral, and pharmacological interventions with childhood central obesity.

**Data Sources:**

MEDLINE, Embase, CINHAL, PsycINFO, PubMed, Academic Search Database, and ProQuest from inception to September 25, 2024.

**Study Selection:**

Inclusion criteria included (1) randomized clinical trials (RCTs) focusing on children aged 5 to 18 years with overweight or obesity at baseline and (2) measured central obesity as a primary or secondary outcome.

**Data Extraction and Synthesis:**

The Preferred Reporting Items for Systematic Reviews and Meta-Analyses reporting guideline was followed. Two authors independently extracted data. Risk of bias was assessed using the revised Cochrane risk-of-bias tool (RoB 2.0). A random-effects meta-analysis was conducted to pool standardized mean differences (SMD) from individual studies. Sensitivity analysis, meta-regression, and subgroup analyses were also conducted.

**Main Outcomes and Measures:**

The main outcome was childhood central obesity measured using waist circumference (WC), waist-to-height ratio, waist-to-hip ratio, and WC *z* score.

**Results:**

This review included 34 RCTs, involving 8183 children aged 5 to 18 years. Twelve studies had a low risk of bias, whereas 8 were deemed to have a higher risk of bias. The meta-analysis of 2 RCTs examining low-fat lunchboxes and a Mediterranean diet along with physical activity (lasting up to 150 minutes per week over 6 to 9 months) showed a significant association with WC (standard mean difference [SMD], −0.38 [95% CI, −0.58 to −0.19]). Five additional RCTs involved behavioral interventions on dietary education to reduce unhealthy snacks, increase fruit and vegetable intake, promote daily physical activity, and limit screen time, supported by online resources also showed a significant association with WC (SMD, −0.54 [95% CI, −1.06 to −0.03]). However, standalone dietary, physical activity, pharmacotherapy, dietary supplements, motivational interviewing, and combined dietary, physical activity, and behavioral methods did not show a significant association with WC.

**Conclusions and Relevance:**

In this meta-analysis of RCTs, combining dietary changes with physical activity, as well as using behavioral strategies alone, were associated with reduced central obesity in children from high- and middle-income countries. Findings from this study have policy implications for Sustainable Development Goals of ending all forms of malnutrition and reducing premature mortality from noncommunicable diseases.

## Key Points

**Question:**

What is the association of physical activity, dietary interventions, supplements, behavioral strategies, and pharmacological treatments, either individually or in combination, with childhood central obesity?

**Findings:**

In this systematic review and meta-analysis of 34 randomized clinical trials with 8183 participants, combining dietary changes with physical activity, as well as standalone behavioral interventions, was associated with significantly reduced central obesity.

**Meaning:**

These results suggest that integrating dietary modifications with physical activity and other behavioral strategies holds promise for reducing central obesity in children.

## Teaser

This systematic review and meta-analysis investigates the association of lifestyle, behavioral, and pharmacological interventions with childhood central obesity.

## Introduction

Childhood central obesity is one of the most serious public health challenges of the 21st century.^1^ In response, the world nations endorsed several strategies to address this global health crisis. The Sustainable Development Goals (SDGs) 2.2 and 3.4 aim to reduce premature mortality from noncommunicable diseases and to end malnutrition in all its forms by 2030, respectively.^2^ The Global Nutrition Target (GNT) 4 aims to stop the increase in childhood obesity by 2025.^3^ Despite these initiatives, globally, the number of school-aged children with obesity increased from 14 million boys and 18 million girls in 1990 to 65 million boys and 94 million girls in 2022.^4^ The World Obesity Federation also estimated that without intervention, the global annual cost of obesity will exceed $4 trillion by 2035.^5^

Preventing central obesity during childhood offers lifelong health benefits and mitigates future health complications.^6^ Central obesity, defined as the excess accumulation of fat in the abdominal area, is a more serious form of obesity.^7^ This is primarily due to increased visceral fat surrounding internal abdominal organs which produce inflammatory cytokines.^8^ Research indicates that while both general and abdominal obesity trends are on the rise globally, the increase in abdominal obesity is often more substantial because of its impact on cardiovascular risk and brain changes in the long term.^9^ For example, a study from China found that the prevalence of abdominal obesity among children increased from 5.0% in 1993 to 19.3% in 2015, a steeper rise compared with the general obesity rates during the same time period.^10^

Body mass index (BMI) is widely used to assess body fat and its associated cardiometabolic risks. However, while BMI is an important metric, it has limitations, particularly in its inability to accurately reflect visceral fat distribution.^11^ To address this, direct measures of central adiposity, such as computed tomography (CT) scans and magnetic resonance imaging (MRI), are considered the criterion standard for assessing visceral fat distribution.^12^ Unfortunately, these methods are costly, time-intensive, and often impractical, particularly in resource-limited settings. Consequently, proxy measures such as waist circumference (WC), waist-to-hip ratio (WHR), and waist-to-height ratio (WHtR) are used to estimate central fat distribution. A previous study found that these anthropometric measures were strongly correlated with all-cause cardiovascular disease (CVD) and cancer mortality, independent of BMI.^13^

Previous systematic reviews have examined the effects of interventions, including physical activity, dietary adjustments, behavioral strategies, and pharmacological approaches, on childhood obesity.^14,15,16,17,18,19,20^ However, these reviews have limitations. Many focused on general obesity rather than specifically targeting central obesity, despite its substantial cardiometabolic risks.^14,21^ Additionally, some studies have narrowed their focus to specific settings (eg, schools, homes, or communities)^14,16,17,19^ or specific intervention types (eg, diet or physical activity).^15,20^ This study conducted a meta-analysis examining lifestyle, behavioral, and pharmacological interventions for childhood central obesity.

## Methods

This systematic review and meta-analysis was reported following the Preferred Reporting Items for Systematic Reviews and Meta-analyses (PRISMA) reporting guideline.^22^ The review is registered in the International Prospective Register of Systematic Reviews (CRD42024541367).

### Eligibility Criteria

The inclusion criteria included: (1) randomized clinical trials (RCTs) comparing physical activity, diet, dietary supplements, behavioral, and pharmacological interventions with either nonintervention, usual care control group, or an alternate intervention; (2) RCTs including children with overweight or obesity aged 5 to 18 years at baseline; and (3) trials measuring WC as primary or secondary outcomes related to central obesity. In studies reporting more than outcome measures (eg, WC and WHtR), we used a predefined hierarchy, prioritizing WC, then WHtR, and if neither was available, other central obesity indices such as WC *z* score. All effect sizes were converted to standardized mean differences (SMDs) to facilitate comparison across studies.

In this review, interventions were categorized as physical activity or dietary when they involved actual physical activity sessions (eg, moderate or vigorous activities) or the provision of dietary items (eg, healthy lunch boxes, grains, fruits, and vegetables), respectively. Meanwhile, any form of health or nutritional education aimed at modifying physical activity, eating habits, or broader lifestyle choices was classified as a behavioral intervention.

RCTs were excluded if participants had any medical or psychiatric conditions at baseline, were using medications affecting weight, or had undergone weight-affecting surgical procedures. Additionally, studies addressing childhood obesity or eating disorders, as well as those solely focused on strength and fitness training, were excluded. Publications not in English were also excluded.

### Information Sources and Search Strategy

Our initial search strategy involved manual checking for published systematic reviews and meta-analyses on interventions for childhood central obesity. We performed a comprehensive search across 6 electronic databases: MEDLINE (Ovid)/PubMed, Embase (Ovid), PsycINFO (EBSCOhost), Academic Search Database (EBSCOhost), ProQuest, and Cumulative Index to Nursing and Allied Health Literature (CINAHL) (EBSCOhost). This search spanned from the inception of the databases until April 15, 2024, for the initial search, and was updated on September 25, 2024. Gray literature was also explored using Google Scholar and Open Dissertation. The search strategy adhered to the Population Intervention Comparator Outcome (PICO) criteria (eTable 1 in Supplement 1).

### Study Selection

The study selection process included 4 steps. First, all articles retrieved from the search databases were exported to EndNote library version 20 (Clarivate Analytics) to remove duplicates.^23^ Second, potentially relevant articles identified during the initial screening underwent full retrieval, and their citations were uploaded to Covidence.^24^ Then, 2 reviewers (S.B.A. and K.Y.A.) independently screened the titles and abstracts of these articles. Third, S.B.A. and K.Y.A. independently conducted a full-text review against the eligibility criteria. Finally, the 2 authors cross-checked their findings to ensure no articles were missed. Any disagreements were resolved through discussion or, if necessary, by involving a third author.

### Data Extraction

The authors (S.B.A. and K.Y.A.) independently extracted data from the included trials using a standardized data extraction form based on the Cochrane Handbook for Systematic Reviews of Interventions. The data collected included information on the authors, year of publication, study design, country of publication, type of intervention, setting of intervention, duration of intervention, number of participants, participants’ age group, control group, and outcomes. The authors (S.B.A. and K.Y.A.) then compared their findings and verified them against the original publications to ensure consistency in the extracted data.

### Risk of Bias Assessment

The revised Cochrane risk-of-bias tool (RoB 2.0) was used to assess the risk of bias in the selected trials.^25^ RoB 2.0 evaluates individual RCTs across 5 domains: bias arising from the randomization process, bias due to deviations from the intended intervention, bias due to missing outcome data, bias in outcome measurement, and bias in the selection of reported results. For cluster RCTs, additional considerations were made for the domain related to the timing of participant identification and recruitment.^26^

### Statistical Analysis

Statistical analyses were conducted using R version 4.3.3 (R Project for Statistical Computing) on the Google Collaboratory platform.^27^ Our initial analysis involved computing mean values and SDs from the reported SEs, 95% CIs, and *P* values using a formula sourced from Cochrane for studies that did not report mean and SD.^28^ Studies reporting effect sizes using different measures were analyzed in standardized mean differences (SMDs). The use of SMD in the meta-analysis allows for the comparison of various outcome measures by converting them into a common metric.^28^ The mean values and SDs provided in the included studies were used to calculate the SMD, ensuring that variations in waist measurement methods did not affect the overall comparability of the results.

Considering the higher level of between-study heterogeneity, a random-effect meta-analysis using an inverse-variance method was used to pool the SMD of individual studies. SMDs and 95% CIs were calculated using the small sample–adjusted SMD, called Hedges *g*.^29^ The Hedges *g* random-effects estimator was also selected for its advantage in accounting for between-study variance.^29^ Between-study heterogeneity was estimated using the *I*^2^ statistic, which indicates the percentage of variability in effect estimates due to heterogeneity rather than sampling error.

We conducted subgroup analyses based on the type of intervention, intervention alone or in combination, intervention settings, and the economic classifications of countries (high income, upper middle income, lower middle income, and low income) as classified by the World Bank.^30^ Further subgroup analyses were performed based on study settings (health care facilities, schools, home, and community levels).

A random-effect meta-regression was conducted using the metafor package in R^31^ to explore the association between study-level characteristics and the effect sizes of central obesity. The covariates included economic region of the country, intervention type, intervention setting, intervention duration, and study design. The maximum likelihood estimation method was used to estimate the residual between-study variance. The Knapp-Hartung variance estimator was applied to calculate the SEs and the 95% CIs.

Publication bias was assessed using a funnel plot and Egger test. All statistical significance thresholds were determined using 2-sided tests, with a *P* < .05 to declare statistical significance. Statistical analysis was performed from September to October 2024.

## Results

### Description of the Studies

**Figure 1.**
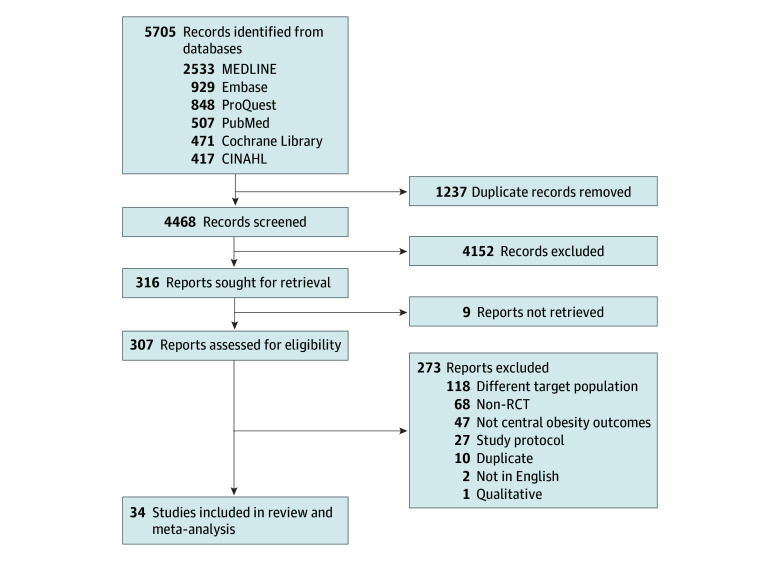
Flow Diagram for the Selection of Eligible Studies RCT indicates randomized clinical trial.

There were 5705 experimental studies identified, from which we excluded 1237 duplicates. During the screening of titles and abstracts, 4152 records were excluded. Following a full-text review of the remaining 316 articles, 34 studies involving 8183 children were included in this study. The remaining 273 studies were excluded for the following reasons: 47 did not focus on central obesity, 68 were non-RCTs, 118 did not focus on children, 27 were trial protocols, 2 were not in English, 1 was qualitative, and 10 were duplicates (Figure 1; eTable 3 in Supplement 1). Out of the 34 eligible trials, 26 were individual RCTs with randomization conducted at the individual level,^32,33,34,35,36,37,38,39,40,41,42,43,44,45,46,47,48,49,50,51,52,53,54,55,56,57^ and the remaining 8 were cluster RCTs where randomization was conducted at the group level (eg, schools).^58,59,60,61,62,63,64,65^ Two trials implemented combined diet and physical activity interventions in schools and homes. The first trial used interventions comprising a low-fat lunchbox, featuring a wide variety of fruits, vegetables, whole grains, lean meats and alternatives, and dairy and dairy alternatives, supported by a 9-month physical activity initiative in schools to ensure students engaged in 150 minutes of sport or class-based activities each week.^59^ The second trial used the Mediterranean diet, with ample vegetables, fresh fruits, olive oil, regular dairy (primarily cheese and yogurt), moderate fish and poultry consumption, limited egg intake (0-4 per week), and reduced red meat. This dietary approach was also supported by 5 supervised exercise sessions each week for 6 months for a total of 120 sessions.^48^

Nine trials combined diet (eg, fruits, vegetables, and grains), physical activity (eg, junior gym sessions, trampolining, and team sports), and behavioral interventions (eg, hour-long health education and consultation lectures by a psychiatrist integrated into the physical activity and dietary programs). Although the content, duration, delivery methods, and comparators varied across the trials, they were conducted over a minimum duration of 3 months and a maximum of 2 years with a mean duration of 10 months. The interventions were implemented in schools, at home, in the community, or at health care facilities.^35,38,40,41,43,51,60,61,65^

Dietary supplementation was also implemented in 5 trials^33,42,47,53,55^ using symbiotic supplementation,^33,47,53^ butyrate, and decaffeinated green tea polyphenols.^55^ Additionally, 2 trials focused solely on physical activity (eg, at least 90 minutes of moderate-to-vigorous physical activity per day on at least 6 days each week).^36,52^ Five trials included exclusively on behavioral interventions focusing on dietary education to reduce unhealthy snacks, increase fruit and vegetable intake, promote daily physical activity, and limit screen time, supported by online resources on healthy lifestyles.^32,49,54,63,64^

One trial diet (eg, whole-grain foods),^46^ one motivational approach (eg, motivation to change eating habits and begin regular physical exercise through the stages of change, the processes of behavioral change, and improved decision-making and self-efficacy),^37^ and another 2 trials of pharmacotherapy (eg, metformin, with its dosage increased weekly from 500 mg/d to 1500 mg/d, and fluoxetine, starting at 10 mg and increasing to 20 mg/d after 3 weeks, and a combination of metformin and fluoxetine^56^ and a 120-mg dose of orlistat 3 times daily for 1 year) were conducted.^39^ One trial combined diet with behavioral interventions (eg, time spent on screen-based recreation each day of the week and consumption of sugar-sweetened beverages).^62^ The remaining 6 trials combined physical activity with behavioral interventions (eg, 2 hours of moderate and vigorous physical activity sessions, warm-up and stretching exercises, jump roping, relay races, stair stepper exercise, handball, table tennis, volleyball, and running, hour-long monthly psychological health education and consultation).^34,44,45,50,57,58^

Twelve trials were conducted in schools,^32,34,45,57,58,59,60,61,62,63,64,65^ while 16 studies in health care facilities.^35,37,38,40,41,42,43,44,46,47,49,52,53,54,55,56^ Most trials were conducted in high-income countries, with no published interventional study on central obesity from low-income countries. Most studies were conducted in Iran,^33,34,46,47,53,56,57,58^ followed by Australia.^44,49,59,62^ The duration of follow-up for trials ranged from 2 to 28 months (eTable 2 in Supplement 1).

### Risk of Bias

Out of 26 individual RCTs, 5 (19%) had a high risk of bias in the randomization process^43,45,47,53,54^ and 4 (15%) had a high risk of bias due to deviations from intended interventions.^41,43,45,47^ Most individual RCTs were also assessed as low risk of bias in the measurement of the outcome^32,33,34,35,36,37,38,40,41,42,44,45,46,47,48,49,50,51,52,53,54,55,56,57^ and missing outcome data.^32,33,34,35,36,37,38,40,41,42,43,44,45,46,47,48,49,50,51,52,53,54,55,56,57^

Overall, a total of 12 individual RCTs (46%) had an overall low risk of bias,^32,33,34,36,37,42,46,49,50,52,56,57^ 6 trials (23%) had some concerns,^35,38,40,44,51,55^ and 8 trials (31%) had a high risk of bias^39,41,43,45,47,48,53,54^ (eFigures 1-4 in Supplement 1). For cluster RCTs, 3 out of 8 studies had a low risk of bias in the randomization process,^59,60,61^ and all studies had a low-risk bias in the selection of reported result.^58,59,60,61,62,63,64,65^ Overall, 2 cluster RCTs (25%) had an overall high risk of bias,^63,65^ and 4 (50%) were assessed as having some concerns.^58,60,62,64^

### Meta-Analysis of Interventions for Central Obesity

**Figure 2.**
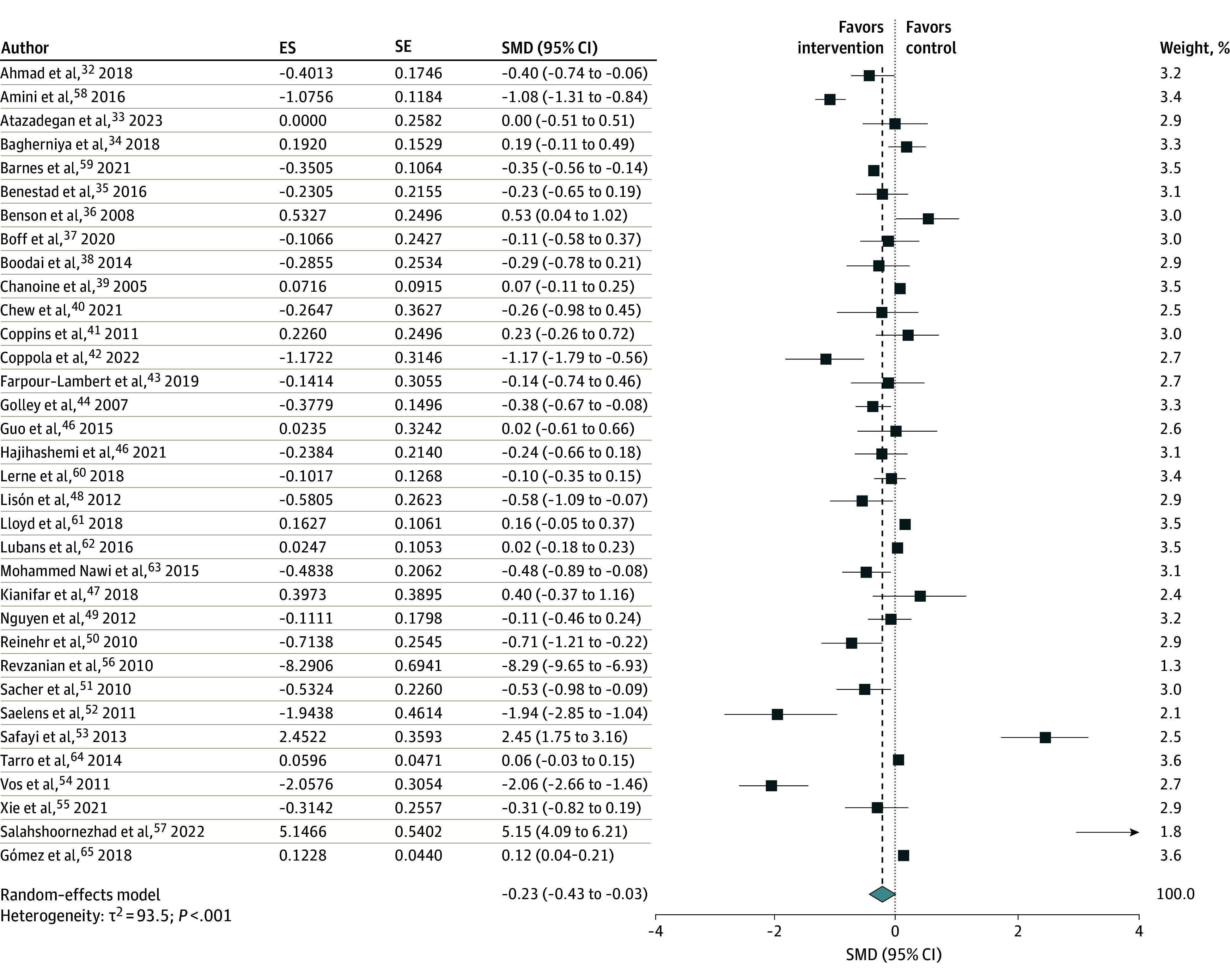
Association of Interventions With Childhood Central Obesity The squares represent the standardized mean difference (SMD) for each study, with the size of the square proportional to the study’s weight in the meta-analysis. Horizontal lines indicate the 95% CI. The diamond at the bottom represents the pooled SMD with its corresponding 95% CI. ES indicates effect size; SE, standard error.

The overall association of the interventions, including dietary, physical activity, behavioral, pharmacological, dietary supplement, and motivational interviewing showed a significant reduction in central obesity (SMD, −0.23 [95% CI, −0.43 to −0.03]; *I*^2^ = 94%) (Figure 2).

**Figure 3.**
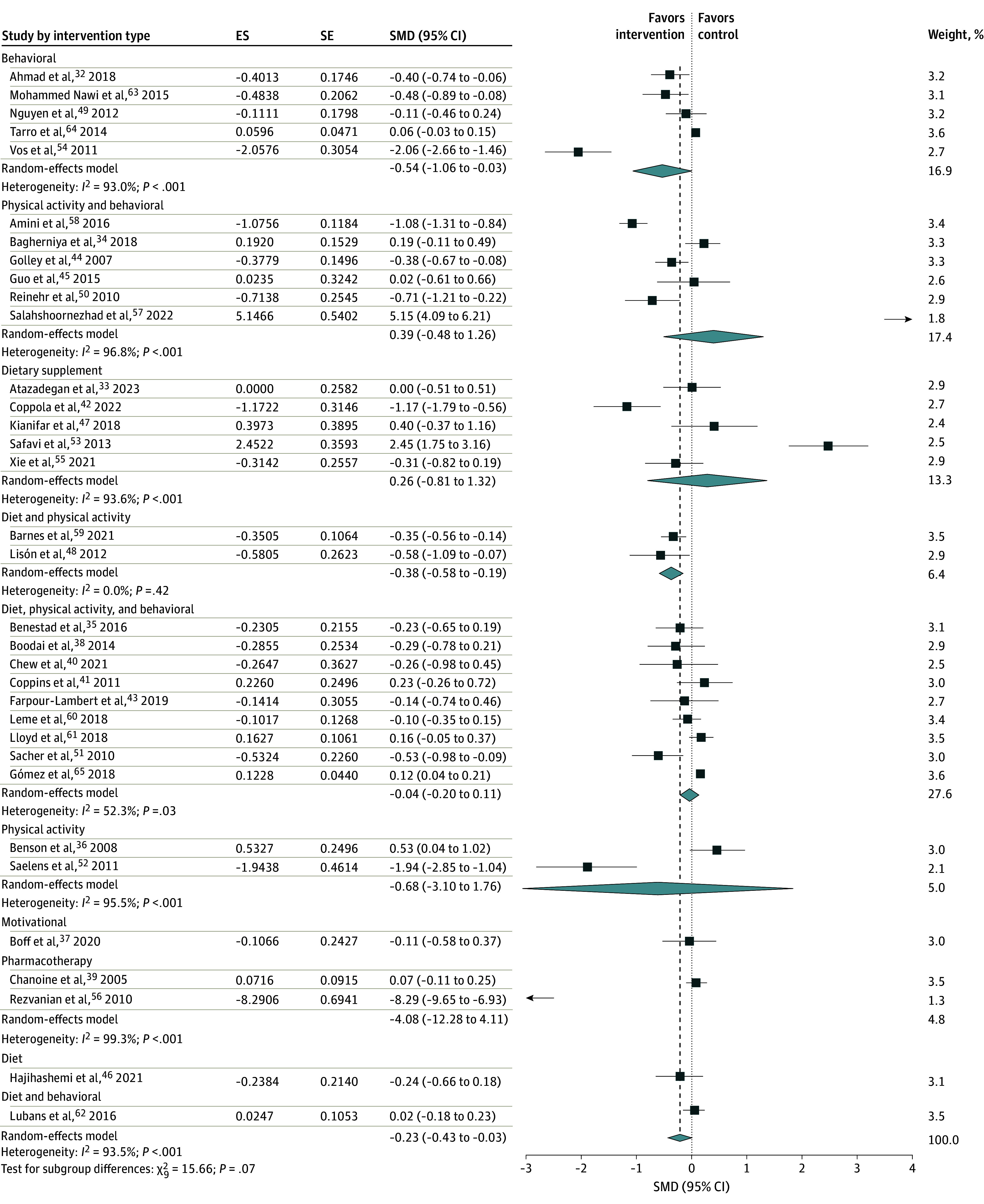
Subgroup Analysis of Interventions With Childhood Central Obesity by Intervention Types The squares represent the standardized mean difference (SMD) for each study, with the size of the square proportional to the study’s weight in the meta-analysis. Horizontal lines indicate the 95% CI. The diamond at the bottom represents the pooled SMD with its corresponding 95% CI. ES indicates effect size; SE, standard error.

Subgroup analyses were conducted based on intervention types, economic regions, trial settings, and study design to address the observed statistical heterogeneity. The meta-analysis of 5 behavioral intervention RCTs found an association with a significant reduction in WC (SMD, −0.54 [95% CI, −1.06 to −0.03]; *I*^2^ = 93%). Additionally, 2 RCTs combining dietary and physical activity interventions were associated with a significant reduction in WC compared with the control group (SMD, −0.38 [95% CI, −0.58 to −0.19]; *I*^2^ = 0%). However, meta-analyses of trials focusing on dietary supplements, as well as standalone interventions such as physical activity, dietary changes, pharmacological treatments, and motivational interviewing, did not show a significant association with WC compared with the control group (Figure 3).

**Figure 4.**
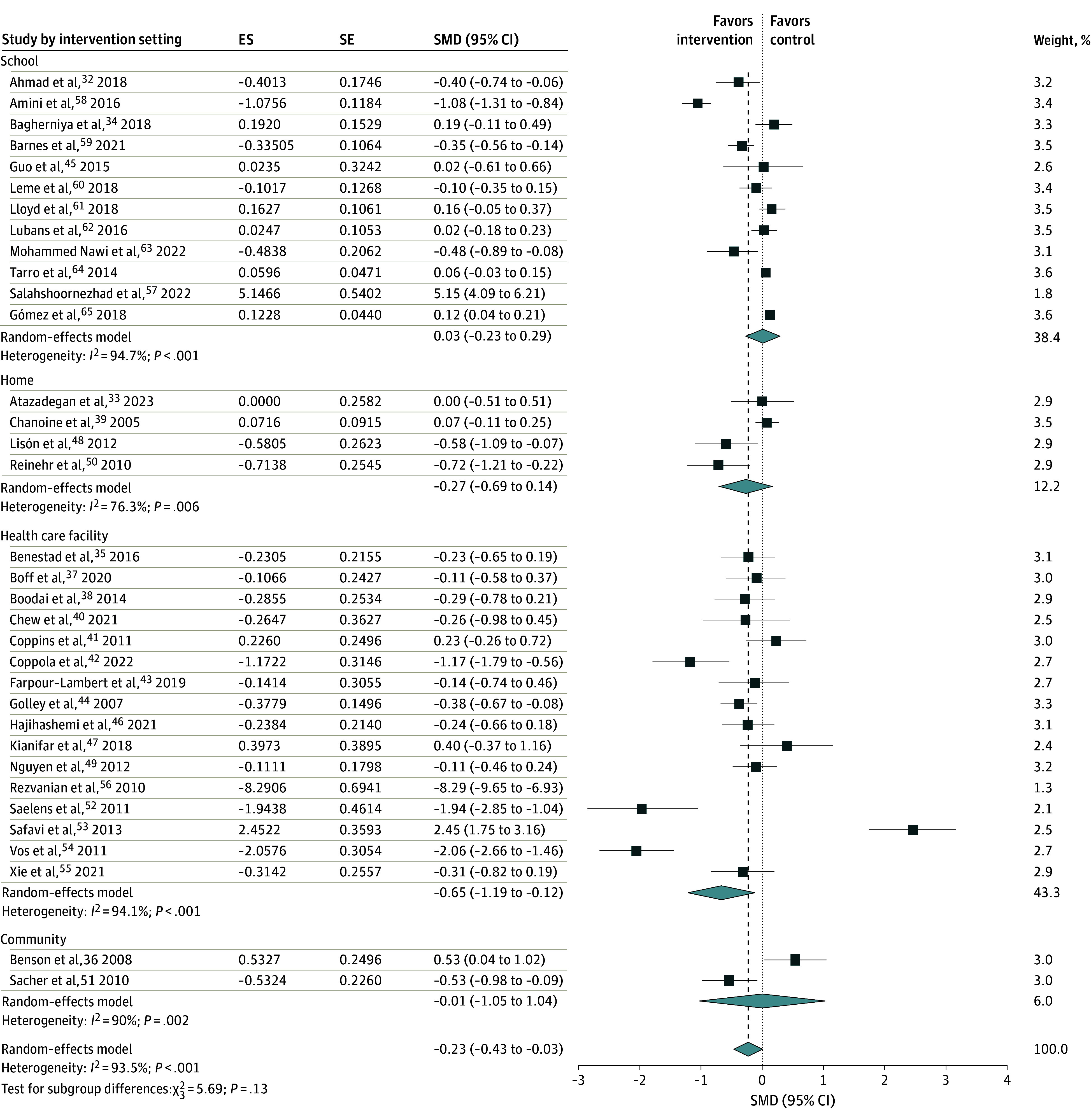
Subgroup Analysis of Interventions With Childhood Central Obesity by Intervention Settings The squares represent the standardized mean difference (SMD) for each study, with the size of the square proportional to the study’s weight in the meta-analysis. Horizontal lines indicate the 95% CI. The diamond at the bottom represents the pooled SMD with its corresponding 95% CI. ES indicates effect size; SE, standard error.

**Figure 5.**
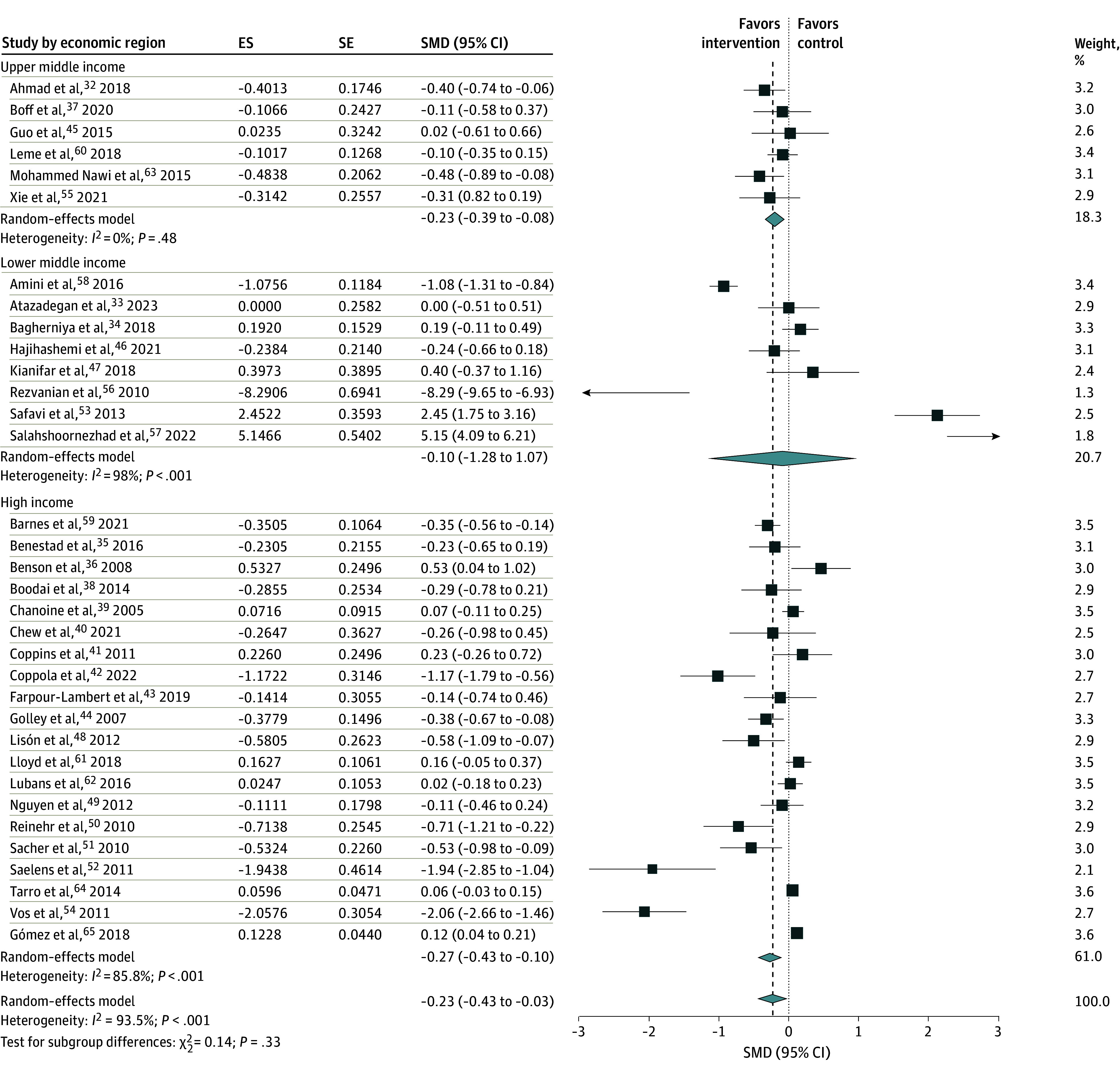
Subgroup Analysis of Interventions With Childhood Central Obesity by Economic Region Of Countries The squares represent the standardized mean difference (SMD) for each study, with the size of the square proportional to the study’s weight in the meta-analysis. Horizontal lines indicate the 95% CI. The diamond at the bottom represents the pooled SMD with its corresponding 95% CI. ES indicates effect size; SE, standard error.

Our subgroup analyses using intervention settings found that 16 RCTs implemented at health care facilities were associated with a significant reduction in WC (SMD, −0.65 [95% CI −1.19 to −0.12]; *I*^2^ = 94%). However, the remaining RCTs implemented at schools, homes, and community levels did not show a significant association with central obesity (Figure 4). Subgroup analysis of trials using a country’s economic status found that 20 trials from high-income countries were associated with a significant reduction in WC (SMD, −0.27 [95% CI, −0.43 to −0.10]; *I*^2^ = 86%). A meta-analysis of 6 trials from upper middle-income countries also showed a significant reduction in WC (SMD, −0.23 [95% CI, −0.39 to −0.08]; *I*^2^ = 0%). However, studies from lower middle-income countries did not show a significant association with WC (SMD, −0.10 [95% CI, −1.28 to 1.07]; *I*^2^ = 98%) (Figure 5).

### Meta-Regression Analysis and Publication Bias

Meta-regression analysis did not reveal a statistically significant association with the overall SMD for WC. Approximately half of the variation in true effect sizes for WC was explained by the included covariates (economic region of the country, intervention type, intervention setting, intervention duration, and study design) in the model (*R*^2^ = 51.90%) (eTable 4 in Supplement 1). Our analysis found no evidence of publication bias, indicated by the visual symmetry of the funnel plots (eFigure 5 in Supplement 1) and the results of Egger test.

## Discussion

To the best of our knowledge, this is the first comprehensive meta-analysis investigating various types of interventions for childhood central obesity. Our findings showed that combining dietary and physical activity interventions was associated with significantly lower central obesity. Similarly, RCTs focusing solely on behavioral interventions significantly reduced central obesity. In contrast, interventions combining dietary, physical activity, and behavioral methods, as well as standalone physical activity, dietary changes, pharmacotherapy, and motivational interviewing, did not show a significant association with reducing central obesity.

RCTs conducted in high-income and upper middle-income countries found a significant reduction in central obesity, whereas trials from lower middle-income countries did not show a significant association with central obesity. Subgroup analyses based on intervention settings showed that interventions implemented at health care facilities were associated with a significant reduction in central obesity.

Previously published systematic reviews have focused on general childhood obesity.^16,18,19,20^ We believe that targeting central obesity during childhood has advantages. First, the global obesity pandemic is rapidly increasing with central obesity being a severe form of the disease.^66^ Second, central obesity is strongly associated with metabolic complications such as diabetes, hypertension, dyslipidemia, and nonalcoholic fatty liver disease, as well as nonmetabolic issues such as weight stigma, bullying, low self-esteem, and depression.^67^ Finally, approximately 55% of children with obesity remain obese into adolescence, and an estimated 80% of adolescents with obesity continue to be obese in adulthood.^68^

Obesity is a complex and multifaceted disease with the interplay of multiple interacting aspects that cannot be addressed with a single intervention.^69^ This is also supported by our findings that combining dietary (eg, fruits, vegetables, and grains) and physical activity (eg, ball games, jogging, and bicycling) interventions, as well as standalone behavioral interventions (eg, hour-long psychological health education and consultation lectures by a psychiatrist integrated into the physical activity and dietary programs) were associated with a significant reduction in central obesity. Tackling childhood obesity and its complications in adulthood requires a whole systems approach across diverse sectors and government agencies.^70^

Additional interventions are also required in the school to implement comprehensive physical activity programs to ensure that children learn healthy habits from a young age. Physical activity offers numerous health benefits, including muscle and bone strength, health and fitness, improved quality of sleep, and ultimately helping to maintain a healthy weight.^71^ At least 1 hour of daily aerobic activity is recommended for children.^71^ This is particularly important for children in high-income countries residing in socioeconomically disadvantaged households where families cannot afford to pay for sports activities. The results of this study also showed that interventions implemented in high-income and upper middle-income countries had a significant association with central obesity. This is potentially because children from developed countries are more likely to achieve recommended dietary and physical activity levels during the intervention period.^72^

### Limitations

This study had limitations. First, the individual studies included in this review used anthropometric measures such as WC, WhtR, WHR, and WC *z* scores to assess central obesity. While these methods are practical and commonly used, particularly in large-scale studies, they are indirect indicators of central adiposity and do not offer the same precision as imaging techniques such as dual-energy x-ray absorptiometry or magnetic resonance imaging.^13^

Second, a considerable level of heterogeneity was detected across the included studies. To address this, we conducted subgroup analyses by intervention types and settings. Third, our review did not identify any RCTs focusing on children with normal BMI but central obesity, despite evidence linking cardiometabolic risks to this group. Fourth, most RCTs were conducted in high- and middle-income countries, limiting the generalizability of our findings to low-income countries.

Fifth, Brown et al^73^ underscored the methodological gaps in global childhood obesity trials, particularly the inadequate handling of clustering effects in RCTs, which risks underestimating standard errors and exaggerating intervention efficacy.^73^ In this meta-analysis, our preliminary review of the included cluster RCTs found that 7 out of 8 studies used statistical methods such as mixed-effects models or generalized estimating equations to adjust for intraclass correlation and design effects. However, 1 study did not clearly report how clustering was addressed. Future cluster RCTs should consider these limitations when designing school-based or other cluster-based trials.

Sixth, some subgroup analyses (eg, standalone physical activity, diet, and combined diet and physical activity interventions) were based on a limited number of studies. These findings should be interpreted cautiously, and future RCTs are recommended to strengthen the evidence on intervention effectiveness. Additionally, there is a chance of publication bias, as studies with negative results are less likely to be submitted for journal publication. We tried to mitigate this by conducting searches of the gray literature.

## Conclusions

Childhood obesity and its cardiometabolic complications in adulthood pose one of the greatest global health challenges of our time, and no country has successfully reversed the global rise in childhood central obesity. In this systematic review and meta-analysis of RCTs on central obesity, combining dietary changes with physical activity, as well as using behavioral strategies alone, showed promise in reducing central obesity in children from high- and middle-income countries. Coordinated efforts from various stakeholders (eg, government, nongovernmental organizations, World Health Organization, Centers for Disease Control and Prevention) will be essential for addressing this multifaceted issue. Findings from this study have policy implications for Sustainable Development Goals 2.2 (ending all forms of malnutrition) and 3.4 (reduce premature mortality from noncommunicable diseases and promote mental health).

## Notes

Supplement 1. **eTable 1.** Databases and Search Strategy **eTable 2.** Description of Included Studies **eTable 3.** Excluded Studies **eTable 4.** Multiple Meta-Regression Model **eFigure 1.** Risk of Bias Traffic Light Plot for Selected Individual RCT Articles **eFigure 2.** Risk of Bias Summary Plot for Selected Individual RCT Articles **eFigure 3.** Risk of Bias Traffic Light Plot for Selected Cluster RCT Articles **eFigure 4.** Risk of Bias Summary Plot for Selected Cluster RCT Articles **eFigure 5.** Funnel Plot of the Included Studies

Supplement 2.
Data Sharing Statement
