# Supplementary material for: Interventions for Childhood Central Obesity: A Systematic Review and Meta-Analysis
Source: JAMA Netw Open. 2025 Apr 11;8(4):e254331. doi: 10.1001/jamanetworkopen.2025.4331 (PMC11992610; doi:10.1001/jamanetworkopen.2025.4331)
Supplement: Supplement 1. — eTable 1. Databases and Search Strategy eTable 2. Description of Included Studies eTable 3. Excluded Studies eTable 4. Multiple Meta-Regression Model eFigure 1. Risk of Bias Traffic Light Plot for Selected Individual RCT Articles eFigure 2. Risk of Bias Summary Plot for Selected Individual RCT Articles eFigure 3. Risk of Bias Traffic Light Plot for Selected Cluster RCT Articles eFigure 4. Risk of Bias Summary Plot for Selected Cluster RCT Articles eFigure 5. Funnel Plot of the Included Studies [file jamanetwopen-e254331-s001.pdf]

Supplemental Online Content

Aychiluhm SB, Mondal UK, Isaac V, Ross AG, Ahmed KY. Interventions for childhood central obesity. *JAMA Netw Open.* 2025;8(4):e254331. doi:10.1001/jamanetworkopen.2025.4331

eTable 1. Databases and Search Strategy

eTable 2. Description of Included Studies

eTable 3. Excluded Studies

eTable 4. Multiple Meta-Regression Model

eFigure 1. Risk of Bias Traffic Light Plot for Selected Individual RCT Articles

eFigure 2. Risk of Bias Summary Plot for Selected Individual RCT Articles

eFigure 3. Risk of Bias Traffic Light Plot for Selected Cluster RCT Articles

eFigure 4. Risk of Bias Summary Plot for Selected Cluster RCT Articles

eFigure 5. Funnel Plot of the Included Studies

This supplemental material has been provided by the authors to give readers additional information about their work.

© 2025 Aychiluhm SB et al. *JAMA Network Open*.

eTable 1: Databases and search strategy

Database	Population	Intervention	Study design	Outcome	
**Medline**	Child/<br>Adolescent/<br>(adolescent or p?ediatric* or school child* or teenage).ab. or youth.af.	Exercise/<br>Diet, Healthy/ or "Diet, Food, and Nutrition"/ or Diet/<br>Physical Fitness/<br>Energy Intake/<br>Health Promotion/<br>Family Therapy/<br>Drug Therapy/<br>Behavior Therapy/<br>Energy Metabolism/<br>Bariatric Surgery/<br>Cognitive Behavioral Therapy/<br>Psychological Techniques/<br>Diet Therapy/<br>((sport or training program or healthy life style or training intervention or physical activity) and exercise program).ab.	Clinical Trial/<br>Randomized Controlled Trials as Topic/<br>Clinical Trials as Topic/<br>Randomized Controlled Trial/<br>Double-Blind Method/<br>Single-Blind Method/<br>Controlled Clinical Trial/<br>(quasiexperimental study or pre-post study or experimental study design or placebo or single arm pre-post study).ab.	Obesity, Abdominal/<br>Obesity/ or Pediatric Obesity/<br>Waist Circumference/<br>Waist-Hip Ratio/<br>Intra-Abdominal Fat/<br>Abdominal Fat/<br>Skinfold Thickness/<br>(central obesity or visceral obesity or visceral adipose tissue or visceral fat).ab.	
**CINAHL**	(MH "Child") OR "age 0-18" OR (MH "Adolescence") OR "pediatric" OR "youth" OR "teenagers"	(MH "Exercise") OR (MH "Physical Activity") OR (MH "Physical Fitness") OR (MH "Life Style, Sedentary") OR (MH "Family Therapy") OR (MH "Drug Therapy, Combination") OR ((MH "Behavior Therapy") OR (MH "Cognitive Therapy")) OR (MH "Bariatric Surgery") OR (MH "Psychological Techniques")	(MH "Randomized Controlled Trials") OR (MH "Clinical Trials") OR (MH "Experimental Studies") OR (MH "Quasi-Experimental Studies") OR (MH "Double-Blind Studies") OR ((MH "Single-Blind Studies") OR (MH "Triple-Blind Studies")) OR "pre-post study"	((MH "Obesity") OR (MH "Pediatric Obesity")) OR ((MH "Waist Circumference") OR (MH "Waist-Hip Ratio")) OR (MH "Adipose Tissue Distribution")	

© 2025 Aychiluhm SB et al. *JAMA Network Open*.

**PubMed**	((((((Child[Title/Abstract]) OR (Adolescent[Title/Abstract])) OR (school child[Title/Abstract])) OR (teenage[Title/Abstract])) OR (youth[Title/Abstract])) OR (pediatric[Title/Abstract])) OR (age 0-18[Title/Abstract])	(((((((((((((Exercise[Title/Abstract]) OR (Diet, Healthy[Title/Abstract])) OR (Physical Fitness[Title/Abstract])) OR (Energy Intake[Title/Abstract])) OR (Health Promotion[Title/Abstract])) OR (Family Therapy[Title/Abstract])) OR (Drug Therapy[Title/Abstract])) OR (Behavior Therapy[Title/Abstract])) OR (Energy Metabolism[Title/Abstract])) OR (Bariatric Surgery[Title/Abstract])) OR (Cognitive Behavioral Therapy[Title/Abstract])) OR (Psychological Techniques[Title/Abstract])) OR (Diet Therapy[Title/Abstract])) OR (sport[Title/Abstract] OR training program[Title/Abstract] OR healthy life style[Title/Abstract] OR training intervention[Title/Abstract] OR physical activity[Title/Abstract] OR exercise program[Title/Abstract])	(((((((((((Clinical Trial[Title/Abstract]) OR (Randomized Controlled Trials as Topic[Title/Abstract])) OR (Clinical Trials as Topic[Title/Abstract])) OR (Randomized Controlled Trial[Title/Abstract])) OR (Double-Blind Method[Title/Abstract])) OR (Single-Blind Method[Title/Abstract])) OR (Controlled Clinical Trial[Title/Abstract])) OR (quasiexperimental study[Title/Abstract])) OR (pre-post study[Title/Abstract])) OR (experimental study design[Title/Abstract])) OR (Single-Blind Studies[Title/Abstract])) OR (Triple-Blind Studies[Title/Abstract])	((((((((((((Obesity[Title/Abstract]) OR (Abdominal[Title/Abstract])) OR (Pediatric Obesity[Title/Abstract])) OR (Waist Circumference[Title/Abstract])) OR (Waist-Hip Ratio[Title/Abstract])) OR (Adipose Tissue Distribution[Title/Abstract])) OR (visceral adipose tissue[Title/Abstract])) OR (Abdominal Fat[Title/Abstract])) OR (Skinfold Thickness[Title/Abstract])) OR (central obesity[Title/Abstract])) OR (visceral obesity[Title/Abstract])) OR (visceral fat[Title/Abstract])) OR (Intra-Abdominal Fat[Title/Abstract])	
**ProQuest**	noft(child OR adolecen* OR youth OR teenager OR pediatric* OR age less than 18)	noft(Exercise OR diet intervention OR physical activ* OR family therapy OR drug therpay OR bariatric therapy OR Behavior Therapy OR Cognitive Therapy OR sport OR training program OR healthy life style OR training intervention)	noft(Randomized Controlled Trial OR clinical trial OR experimental studies OR quasi experimental studies OR double-blind studies OR pre-post study)	noft(central obesity OR abdominal obesity OR visceral fat accumulation OR Waist Circumference OR Waist-Hip Ratio)	Limited by: Language:English Narrowed by:Full text: Full text; Peer reviewed: Peer reviewed

© 2025 Aychiluhm SB et al. *JAMA Network Open*.

**Cochrane**	#1MeSH descriptor: [Child] explode all trees<br>#2(adolesent):ti,ab,kw OR (pediatric):ti,ab,kw OR (youth):ti,ab,kw OR (teenagers):ti,ab,kw	#4MeSH descriptor: [Exercise] explode all trees<br>#5("Bariatric Surgery"):ti,ab,kw OR ((diet NEXT therapy*)):ti,ab,kw OR ((family NEXT therapy*)):ti,ab,kw OR ("drug therapy"):ti,ab,kw OR ((behavior NEXT therapy*)):ti,ab,kw	#7MeSH descriptor: [Randomized Controlled Trial] explode all tree<br>#8("clinical trial"):ti,ab,kw OR ("experimental studies"):ti,ab,kw OR ("quasi experimental studies"):ti,ab,kw OR ("double-blind studies"):ti,ab,kw OR ("pre-post study"):ti,ab,kw	#10MeSH descriptor: [Obesity] explode all trees<br>#11("central obesity"):ti,ab,kw OR ("abdominal obesity"):ti,ab,kw OR ("visceral fat accumulation"):ti,ab,kw OR ("Waist Circumference"):ti,ab,kw OR ("Waist-Hip Ratio"):ti,ab,kw	Limited by: Language:English
**Embase**	child/ or school child/ OR adolescent/ or juvenile/ OR (adolescent or p?ediatric or school child* or teenage).ab.	exercise/<br>OR diet/ or dash diet/ or healthy OR diet/ or mediterranean diet/ or unhealthy diet/OR<br>fitness/ OR<br>family therapy/ or parent-child interaction therapy/ OR<br>drug therapy/ OR<br>behavior therapy/ OR<br>bariatric surgery/ OR<br>motivational interviewing/ or counseling/ OR<br>sport or training program or healthy life style or<br>training intervention or physical activity or exercise program).ab.	clinical trial/ or controlled clinical trial/ or multicenter study/ or phase 1 clinical trial/ or phase 2 clinical trial/ or phase 3 clinical trial/ or phase 4 OR<br>clinical trial/ OR<br>"randomized controlled trial (topic)"/ OR<br>double blind procedure/ OR<br>quasi experimental study/ OR<br>(quasi-experimental study or pre-post study or experimental study or placebo).ab.	abdominal obesity/ OR<br>childhood obesity/ OR<br>skinfold thickness/ OR<br>waist circumference/ OR<br>waist hip ratio/ OR<br>intra-abdominal fat/ OR<br>(central obesity or visceral obesity or visceral adipose tissue or visceral fat).ab.	

© 2025 Aychiluhm SB et al. *JAMA Network Open*.

eTable 2: Description of included studies.

Author, Year	Country	Age group	Intervention types	Intervention Setting	Total sample size at the end of the intervention	Duration of intervention	Outcome Measures Used
Ahmad (2018)	Malaysia	8-11 years	**Interventions (Behavioural)**<br>Dietary and physical activity (PA) education on:<br>• Reducing sugar-sweetened beverages and unhealthy snacks<br>• Consuming at least five servings of fruits and vegetables daily<br>• 30+ minutes of moderate to vigorous activities daily<br>• Limiting screen daily time to a maximum of 120 minutes<br>**Control**<br>Wait-list control group	School	134	4 months	Waist Circumference (WC)
Amini (2016)	Iran	Not reported	**Interventions (PA and Behavioural)**<br>Nutritional, lifestyle, and PA education on:<br>• Food groups, energy, and obesogenic situations and strategies to overcome them<br>• Increase physical activity of pupils indoor and outdoor<br>• Improvement of school canteens to have a healthy diet<br>Physical activity sessions<br>• Two hours of moderate and vigorous PA sessions, warm-up and stretching exercises, jump roping, relay races, stair stepper exercise, handball, table tennis, volleyball, and running<br>**Control**<br>No intervention	School	327	4.5 months	WC
Atazadegan (2023)	Iran	8-18 years	**Intervention (Dietary supplements)**<br>• Symbiotic (prebiotic and probiotic dietary supplementations)<br>**Control**<br>• Placebo	Home	60	2 months	WC

© 2025 Aychiluhm SB et al. *JAMA Network Open*.

Bagherniya (2018)	Iran	12-16 years	**Interventions (PA and Behavioural)**<br>• Sports workshops and counselling sessions for students, parents, and teachers<br>• SMS text messages for students and parents and<br>• Free practical and competitive sports sessions for 180 minutes per week<br>**Control**<br>Self-help handbooks, and usual physical education sessions	School	172	7.5 months	WC
Barnes (2021)	Australi a	5-12 years	**Interventions 1 (PA):**<br>• A 9-month physical activity intervention at schools to deliver 150 minutes each week via physical education, sport, or class-based activities<br>**Intervention 2 (Diet):**<br>• A healthy school lunchbox (wide variety of fruit, vegetables, grain (cereal) foods, lean meats and meat alternatives, and dairy and dairy alternatives)<br>**Intervention 3 (Diet and PA):**<br>• Concurrent provision of both diet and PA interventions<br>**Control**<br>• Usual practices without any PA and dietary support	School	362	9 months	WC
Benestad (2016)	Norway	7-12 years	**Intervention (summer camp Dietary, PA, and Behavioural):**<br>• Lifestyle education for children<br>• Engage in ≥ 1 hour of physical activity a day and limit screen time to ≤ 2 hours a day<br>• Reducing sugary drinks, eating breakfast, eating at home, eating ≥ 5 portions of fruit or vegetables a day, whole grain products, fish, lean meats, sharing at least 5-6 family meals a week, self-regulation of meals<br>**Control (lifestyle school group):**<br>• Four outpatient days in a tertiary care hospital	Healthcar e Facility	87	2 years	WC
Benson (2008)	New Zealand	7-13 years	**Interventions (PA):**<br>• Supervised high-intensity resistance training (upper and lower body exercise training) using	Communit y	67	2 months	WC

© 2025 Aychiluhm SB et al. *JAMA Network Open*.

			free weights (dumbbells and bars) and weighted ankle cuffs. Each training session included a set of 11 exercises aimed at targeting all major muscle groups, performed at high intensity.<br>**Control**:<br>• Wait-list control group				
Boff (2020)	Brazil	16-18 years	**Interventions (Motivational interviewing):**<br>• Twelve 1.5-hour weekly meetings over three months, based on the Transtheoretical Model (TTM), aimed to motivate changes in eating habits, promote regular exercise and enhance decision-making and self-efficacy through the stages and processes of behavioural change.<br>**Control**<br>• Traditional health education was provided for the same number of sessions	Healthcare Facility	68	3 months	WC
Boodai (2014)	Kuwait	10-14 years	**Interventions (Dietary, PA, and Behavioural):**<br>• Reducing sedentary behaviour and screen time, with discussions on the pros and cons of lifestyle changes, including diet and physical activity.<br>• Goal setting about diet, physical activity, and sedentary behaviour; and relapse prevention.<br>• Dietary measures using a modified version of the 'traffic light diet' system<br>• Walking and sport activities<br>**Control**<br>• Referral to primary care	Healthcare Facility	63	6 months	WC
Chanoine (2005)	United States & Canada	12-16 years	**Interventions (Pharmacological):**<br>• A 120-mg dose of orlistat 3 times daily for 1 year<br>**Control**<br>• Placebo identical capsules 3 times daily for 1 year	Home	533	12 months	WC
Chew (2021)	Singapore	10-16 years	**Interventions (Dietary, PA, and Behavioural)**<br>• LITE (Lifestyle Intervention for TEenagers) group programme<br>• Adopting healthy food choices & eating patterns, increasing physical activity, & reducing sedentary behaviours	Healthcare Facility	31	6 months	WC

© 2025 Aychiluhm SB et al. *JAMA Network Open*.

			• Vigorous, moderate, and walking physical activities<br>• Improving fruit and vegetable consumption<br>**Control**<br>• Usual care				
Coppins (2011)	United Kingdom	6-14 years	**Interventions (Dietary, PA, and Behavioural)**<br>• Two Saturday morning workshops (8 h in total) held 1–2 weeks focusing on healthy eating, physical activity, reducing sedentary behaviour, behaviour change, and psychological well being<br>• Two physical activity sessions of 1 h/week led by physical activity instructors and included junior gym sessions (bikes and various weights), circuits, trampolining, rock climbing, table tennis, basketball, tennis, badminton, football, and the bleep test.<br>**Control**<br>• Wait-list control group	Healthcare Facility	65	12 months	WC
Coppola (2022)	Italy	5-17 years	**Interventions (Dietary supplement):**<br>• Oral sodium butyrate, 20 mg/kg body weight per day for 6 months<br>**Control**<br>• Placebo capsules	Healthcare Facility	48	6 months	WC
Farpour-Lambert (2019)	Switzerland	7.5-11.9 years	**Interventions 1 (Moderate intensity Dietary, PA, and Behavioural)**<br>• Seven monthly 60-minute sessions promoting low-saturated fat, nutrient-dense foods; physical activity, stairs, leisure activities, sports, and reduced sedentary behaviour<br>• Self-awareness, problem-solving, goal-setting, stimulus-control, coping-skills, empowerment, parental guidance, and relapse prevention.<br>**Intervention 2 (High-intensity Dietary, PA, and Behavioural)**<br>• 14 sessions (11 weekly then 3 monthly meetings) over 6 months. The sessions are similar with moderate intensity.<br>**Control**	Healthcare Facility	43	12 months	WC

© 2025 Aychiluhm SB et al. *JAMA Network Open*.

			• Standard care				
Golley (2007)	Australi a	6- 9 years	**Interventions (PA and Behavioural)**<br>• Parenting skills training helped parents implement family lifestyle changes.<br>• Intensive lifestyle support focused on healthy eating, portion control, activity, and self-esteem.<br>• Structured, supervised activities designed by physical activity experts.<br>**Control**<br>• Wait-listed control	Healthcar e Facility	182	12 months	WC z score
Gomez (201 8)	Spain	8-10 years	**Interventions (Dietary, PA, and Behavioural)**<br>• Workshops about eating habits and cooking techniques by a nutrition expert.<br>• Mediterranean Diet, to be implemented by restaurants, food markets, and local food producers.<br>• **M**ountain hikes, activities at public parks, family races, etc., to be implemented by schools, parent associations, and public libraries<br>**Control**<br>• Standard care	School	2080	15 months	Waist-to-height ratio (WHtR)
Guo (2015)	China	not reported	**Interventions (PA, and Behavioural)**<br>• Monthly 40-minute nutrition lessons followed by 20 minutes of interaction.<br>• 60 minutes of moderate to vigorous aerobic activity.<br>• Hour-long monthly psychological health education and consultation for intervention students.<br>**Control**<br>• Standard care	School	41	12 months	WC
Hajihashemi (2021)	Iran	8-15 years	**Interventions (Diet):**<br>• The required servings of grains and other food groups for each participant was calculated based on a macronutrient ratio of 53% carbohydrates, 30% fats, and 17% proteins.<br>• Participants were provided the specific number of grain servings (e.g., brown rice, whole-meal biscuits, bulgur, barley bread, popcorn) needed	Healthcar e Facility	88	2 months	WC

© 2025 Aychiluhm SB et al. *JAMA Network Open*.

			**Control:**<br>• Individuals in the non-intervention group were asked not to consume any of these foods				
Kianifar (2018)	Iran	7-13 years	**Interventions (Dietary supplement):**<br>• A symbiotic capsule (composed of prebiotics, vitamin A, C, and E, and Lactobacillus casei, L rhamnosus, Streptococcus thermophilus, Bifidobacterium breve, L acidophilus, B infantis, and L bulgaricus) per day for 12 weeks<br>**Control:**<br>• **Placebo**	Healthcare Facility	27	3 months	WC
Leme (2018)	Brazil	14-18 years	**Interventions (Dietary, PA, and Behavioural)**<br>• Eat fruit and vegetables every day<br>• Warmups and strengths and resistance training<br>• Reduce your sitting time during recess, after school and on weekends<br>**Control**<br>• No interventions	School	253	6 months	WC
Lison (2012)	Spain	6-16 years	**Interventions (Dietary and PA)**<br>• Promotion of the Mediterranean diet: plenty of vegetables, fresh fruit, olive oil, regular dairy (mainly cheese and yogurt), moderate fish and poultry, 0-4 eggs weekly, and reduced red meat.<br>• Five supervised exercise sessions per week for 6 months (120 sessions)<br>**Control**<br>• No interventions	Home	65	6 months	WC
Lloyd (2018)	United Kingdom	9-10 years	**Interventions (Dietary, PA, and Behavioural)**<br>• Reduce sugar-sweetened beverages.<br>• Physical activity on at least 3 weekdays and 1 weekend day, 10 hours each, including football, games, and dances.<br>• Promotion program emphasized a healthy lifestyle, focusing on sugar-sweetened drinks, snacking, physical activity, and reduced screen time, promoting the 80/20 rule for balanced living.<br>**Control**	School	359	3 months	WC z score

© 2025 Aychiluhm SB et al. *JAMA Network Open*.

			• Standard education provision				
Lubans (2016)	Australia	12-14 years	**Interventions (Diet and Behavioural):**<br>• The intervention was based on Self-Determination Theory and Social Cognitive Theory and involved: professional development, fitness equipment for schools, teacher-delivered physical activity sessions, lunch-time activity sessions, researcher-led seminars, a smartphone application, and parental strategies.<br>**Control**<br>• Wait-listed control	School	361	5 months	WC
MohammedNawi (2015)	Malaysia	16 years	**Interventions (Behavioural):**<br>• Internet-based information on healthy lifestyles, diet, and ways to overcome obesity.<br>**Control**<br>• Printed reading materials	School	97	3 months	WC
Nguyen (2012)	Australia	13-16 years	**Interventions (Behavioural):**<br>• Loozit® group program: cognitive behavioural therapy for parents and adolescents using behavioural principles to change dietary intake and activity levels, and social cognitive approaches to modify self-efficacy, motivation, perseverance, and self-regulation<br>• The intervention groups received additional telephone coaching and electronic communications<br>**Control**:<br>• Loozit® group program	Healthcare Facility	124	12 months	WC
Reinehr (2010)	German	8-16 years	**Interventions (PA and Behavioural):**<br>• Physical activity training once a week (1.5 h per session) for 6 months and consisted of ball games, jogging, trampoline jumping, dancing for girls, wrestling for boys, and instructions in physical activity as part of everyday life, as well as in reduction of the amount of time spent watching television or playing computer games.	Home	66	6 months	WC

© 2025 Aychiluhm SB et al. *JAMA Network Open*.

			• Nutritional course (using a “traffic-light system” for food selection) and in the eating behaviour course<br>**Control**<br>• No intervention				
Rezvanian (2010)	Iran	10-18 years	**Interventions 1 (Pharmacotherapy):**<br>• Metformin 500 mg/day to 1500 mg/day.<br>**Interventions 2 (Pharmacotherapy):**<br>• Fluoxetine, with the initial dosage of 10 mg and increased to 20 mg/day after 3 weeks<br>**Interventions 2 (Pharmacotherapy):**<br>• A combination of Metformin and Fluoxetine<br>**Control**<br>• Placebo	Healthcare Facility	83	4 months	WC
Sacher (2010)	United Kingdom	8-12 years	**Interventions (Dietary, PA and Behavioural):**<br>• Nutrition education sessions, including providing healthy recipes for families to try at home.<br>• Hands-on sessions involving the preparation of healthy meals and the sampling of fruits and vegetables.<br>• Instruct parents and children on applying behavioural techniques, such as stimulus control, goal setting, reinforcement, and response prevention.<br>• Each session also included one hour of exercise for the children.<br>**Control**<br>• Wait-list control	Community	82	6 months	WC
Saelens (2011)	United States	7-11 years	**Interventions (PA):**<br>• At least 90 minutes of moderate-to-vigorous PA per day on at least 6 days each week. (additional 30 minutes of PA compared to the control)<br>**Control**<br>• At least 60 minutes of moderate-to-vigorous PA per day on at least 6 days each week.	Healthcare Facility	29	4 months	WC
Safavi (2013)	Iran	6-18 years	**Interventions (Synbiotic dietary supplements):**<br>• Each capsule contained a blend of freeze-dried Lactobacillus and Bifidobacterium strains (2.0 ×	Healthcare Facility	56	2 months	WC

© 2025 Aychiluhm SB et al. *JAMA Network Open*.

			10⁸ CFU), prebiotics (fructo-oligosaccharides), and vitamins E, A, and C. Participants took one capsule daily before a main meal for 8 weeks.<br>**Control**<br>• Placebo				
Salahshoorn ezhad (2022)	Iran	9-12 years	**Interventions (PA and Behavioural):**<br>• A smartphone game-based nutrition education aimed at improving food choices by reducing high-calorie foods and increasing fibre, fruits, vegetables, whole grains, legumes, and low-fat dairy.<br>• Cognitive behavioral therapy sessions.<br>• Three 45-minute aerobic exercise sessions per week for ten weeks.<br>**Control**<br>• Standard weigh control training	School	62	3 months	WC
Tarro (2014)	Spain	7-8 years	**Interventions (Behavioural):**<br>• Educational intervention on eight lifestyles (1) to improve healthy lifestyle; 2) to encourage healthy drinks intake (and avoidance of unhealthy carbonated/sugared beverages); 3) to increase vegetables and legumes consumption; and 4) to decrease candies and pastries while increasing the intake of fresh fruits and nuts; 5) to improve healthy habits within a set timetable (home meals, teeth-brushing, hand-washing) and physical activity participation; 6) to increase fruit intake; 7) to improve dairy product consumption; and 8) to increase fish consumption.<br>**Control**<br>• Standard intervention	School	1939	28 months	WC
Vos (2011)	Netherlands	8-17 years	**Interventions (Behavioural):**<br>• Nutritional advice using traffic-light lists for major food groups.<br>• Physical activity counselling.<br>• Psychological counselling through motivational interviewing.<br>**Control**	Healthcare Facility	67	12 months	WC z score

© 2025 Aychiluhm SB et al. *JAMA Network Open*.

			• Standard care and advice				
Xie (2021)	China	6-10 years	**Interventions (Dietary supplements):**<br>• Decaffeinated Green Tea Polyphenols (DGTP) 400 mg/day<br>**Control**<br>• **Placebo**	Healthcare Facility	62	3 months	WC

© 2025 Aychiluhm SB et al. *JAMA Network Open*.

eTable 3: Excluded studies

S.N	Title	Reason for exclusion
1	Aadland E, Nilsen AK, Haugland ES, Vabø KB, Aadland KN. The multivariate physical activity signatures associated with body mass index and waist-to-height ratio in 3–5-year-old Norwegian children. Preventive Medicine Reports. 2022 Oct 1;29:101930.	Non-RCT
2	Adab P, Pallan MJ, Cade J, Ekelund U, Barrett T, Daley A, Deeks J, Duda J, Gill P, Parry J, Bhopal R. Preventing childhood obesity, phase II feasibility study focusing on South Asians: BEACHeS. BMJ Open, 4 (4), e004579.	Non-RCT
3	Adab, P., Pallan, M.J., Lancashire, E.R. et al. A cluster-randomised controlled trial to assess the effectiveness and cost-effectiveness of a childhood obesity prevention programme delivered through schools, targeting 6–7 year old children: the WAVES study protocol. BMC Public Health 15, 488 (2015). https://doi.org/10.1186/s12889-015-1800-8.	study protocol
4	Alemayehu HK, Salvadego D, Isola M, Tringali G, De Micheli R, Caccavale M, Sartorio A, Grassi B. Three weeks of respiratory muscle endurance training improve the O2 cost of walking and exercise tolerance in obese adolescents. Physiological reports. 2018 Oct;6(20):e13888.	No central obesity outcome
5	Alexandrou C, Henriksson H, Henström M, Henriksson P, Delisle Nyström C, Bendtsen M, Löf M. Effectiveness of a smartphone app (MINISTOP 2.0) integrated in primary child health care to promote healthy diet and physical activity behaviors and prevent obesity in preschool-aged children: randomized controlled trial. International Journal of Behavioral Nutrition and Physical Activity. 2023 Feb 21;20(1):22.	Different target population
6	Di Maglie A, Marsigliante S, My G, Colazzo S, Muscella A. Effects of a physical activity intervention on schoolchildren fitness. Physiological reports. 2022 Jan;10(2):e15115.	Different target population
7	Aragón-Martín R, Gomez-Sanchez MD, Jiménez-Pavón D, Martínez-Nieto JM, Schwarz-Rodríguez M, Segundo-Iglesias C, Novalbos-Ruiz JP, Santi-Cano MJ, Castro-Piñero J, Lineros-González C, Hernán-García M. A Multimodal Intervention for Prevention of Overweight and Obesity in Schoolchildren. A Protocol Study “PREVIENE-CÁDIZ”. International journal of environmental research and public health. 2021 Feb;18(4):1622.	study protocol
8	Boudreau AD, Kurowski DS, Gonzalez WI, Dimond MA, Oreskovic NM. Latino families, primary care, and childhood obesity: a randomized controlled trial. American journal of preventive medicine. 2013 Mar 1;44(3):S247-57.	No central obesity outcome
9	Arce B, Davis-Ajami ML, Ortyl B, Hartlieb K, Dickinson S, Golzarri-Arroyo L, Olcott C, Faith M, Jayawardene W. 'eatNplay'A Family-Based Telehealth Intervention for Childhood Obesity: A Mixed-Methods RCT Study. Obesity. 2022 Nov 1;30:55-.	Reports not retrieved.
10	Ariza C, Sánchez-Martínez F, Serral G, Valmayor S, Juárez O, Pasarín MI, Castell C, Rajmil L, López MJ, POIBA Project Evaluation Group. The Incidence of obesity, assessed as adiposity, is reduced after 1 year in primary schoolchildren by the POIBA intervention. The Journal of nutrition. 2019 Feb 1;149(2):258-69.	Non-RCT
11	Bacardí-Gascon M, Jiménez-Cruz A. A six month randomized school intervention and an 18-month follow-up intervention to prevent childhood obesity in Mexican elementary schools. Nutricion Hospitalaria. 2012;27(3):755-62.	Non-RCT
12	Bäcklund C, Sundelin G, Larsson C. Effects of a 2-year lifestyle intervention on physical activity in overweight and obese children. Advances in physiotherapy. 2011 Sep 1;13(3):97-109.	No central obesity outcome

© 2025 Aychiluhm SB et al. *JAMA Network Open*.

13	Woo Baidal JA, Nelson CC, Perkins M, Colchamiro R, Leung-Strle P, Kwass JA, Gortmaker SL, Davison KK, Taveras EM. Childhood obesity prevention in the Women, Infants, and Children Program: outcomes of the MA-CORD study. Obesity. 2017 Jul;25(7):1167-74.	Different target population
14	Baltaci A, Hurtado Choque GA, Davey C, Reyes Peralta A, Alvarez de Davila S, Zhang Y, Gold A, Larson N, Reicks M. Padres Preparados, Jóvenes Saludables: intervention impact of a randomized controlled trial on Latino father and adolescent energy balance-related behaviors. BMC public health. 2022 Oct 18;22(1):1932.	Different target population
15	Baranowski T, Baranowski J, Chen TA, Buday R, Beltran A, Dadabhoy H, Ryan C, Lu AS. Videogames that encourage healthy behavior did not alter fasting insulin or other diabetes risks in children: randomized clinical trial. Games for Health Journal. 2019 Aug 1;8(4):257-64.	Different target population
16	Barbeau P, Johnson MH, Howe CA, Allison J, Davis CL, Gutin B, Lemmon CR. Ten months of exercise improves general and visceral adiposity, bone, and fitness in black girls. Obesity. 2007 Aug;15(8):2077-85.	Different target population
17	Baria F, Kamimura MA, Aoike DT, Ammirati A, Leister Rocha M, de Mello MT, Cuppari L. Randomized controlled trial to evaluate the impact of aerobic exercise on visceral fat in overweight chronic kidney disease patients. Nephrology Dialysis Transplantation. 2014 Apr 1;29(4):857-64.	Different target population
18	Barkin SL, Gesell SB, Póe EK, Ip EH. Changing overweight Latino preadolescent body mass index: the effect of the parent-child dyad. Clinical pediatrics. 2011 Jan;50(1):29-36.	No central obesity outcome
19	Bartelink NH, van Assema P, Kremers SP, Savelberg HH, Oosterhoff M, Willeboordse M, van Schayck OC, Winkens B, Jansen MW. Can the Healthy Primary School of the Future offer perspective in the ongoing obesity epidemic in young children? A Dutch quasi-experimental study. BMJ open. 2019 Oct 1;9(10):e030676.	Non-RCT
20	Beaulieu D, Godin G. Staying in school for lunch instead of eating in fast-food restaurants: results of a quasi-experimental study among high-school students. Public health nutrition. 2012 Dec;15(12):2310-9.	Non-RCT
21	Berntsen S, Mowinckel P, Carlsen KH, Lødrup Carlsen KC, Pollestad Kolsgaard ML, Joner G, Anderssen SA. Obese children playing towards an active lifestyle. International Journal of Pediatric Obesity. 2010 Jan 1;5(1):64-71.	No central obesity outcome
22	Colindres M, Vu MB, Davis LP. Latino caregiver's insight into childhood overweight management and relationships with their health care providers. Hispanic Health Care International. 2009;7(1).	Qualitative study
23	Bharath LP, Choi WW, Cho JM, Skobodzinski AA, Wong A, Sweeney TE, Park SY. Combined resistance and aerobic exercise training reduces insulin resistance and central adiposity in adolescent girls who are obese: randomized clinical trial. European journal of applied physiology. 2018 Aug;118:1653-60.	Different target population
24	Bhave S, Pandit A, Yeravdekar R, Madkaikar V, Chinchwade T, Shaikh N, Shaikh T, Naik S, Marley-Zagar E, Fall CH. Effectiveness of a 5-year school-based intervention programme to reduce adiposity and improve fitness and lifestyle in Indian children; the SYM-KEM study. Archives of Disease in Childhood. 2016 Jan 1;101(1):33-41.	Different target population
25	Bibiloni MD, Fernández-Blanco J, Pujol-Plana N, Pujol-Puyané MC, Tur JA. Reversion of overweight and obesity in Vilafranca del Penedes child population: ACTIVA'T Program (2012). Gaceta sanitaria. 2017 Nov 21;33(2):197-202.	Non-RCT
26	Bocca G, Corpeleijn E, Stolk RP, Sauer PJ. Results of a multidisciplinary treatment program in 3-year-old to 5-year-old overweight or obese children: a randomized controlled clinical trial. Archives of Pediatrics & Adolescent Medicine. 2012 Dec 1;166(12):1109-15.	Different target population
27	Bocca G, Corpeleijn E, van den Heuvel ER, Stolk RP, Sauer PJ. Three-year follow-up of 3-year-old to 5-year-old children after participation in a multidisciplinary or a usual-care obesity treatment program. Clinical nutrition. 2014 Dec 1;33(6):1095-100.	Duplicate
28	Booth ML, Macaskill P, Lazarus R, Baur LA. Sociodemographic distribution of measures of body fatness among children and adolescents in New South Wales, Australia. International Journal of Obesity. 1999 May;23(5):456-62.	Different target population

© 2025 Aychiluhm SB et al. *JAMA Network Open*.

29	Borfe L, Brand C, Schneiders LD, Mota J, Cavaglieri CR, Leite N, Renner JD, Reuter CP, Gaya AR. Effects and responsiveness of a multicomponent intervention on body composition, physical fitness, and leptin in overweight/obese adolescents. International journal of environmental research and public health. 2021 Jul 7;18(14):7267.	Non-RCT
30	Boutelle KN, Rhee KE, Liang J, Braden A, Douglas J, Strong D, Rock CL, Wilfley DE, Epstein LH, Crow SJ. Effect of attendance of the child on body weight, energy intake, and physical activity in childhood obesity treatment: a randomized clinical trial. JAMA pediatrics. 2017 Jul 1;171(7):622-8.	No central obesity outcome
31	ALBUQUERQUE FILHO NB, Bellaguarda ER, Rebouças GM, Felipe TR, Dantas PM, Knackfuss MI, Medeiros HJ. Concurrent exercise program plus diet intervention on body adiposity and lipid profile in obese adolescents. GAZZETTA MEDICA ITALIANA-ARCHIVIO PER LE SCIENZE MEDICHE. 2015;174(6):259.	Different target population
32	Brandstetter S, Klenk J, Berg S, Galm C, Fritz M, Peter R, Prokopchuk D, Steiner RP, Wartha O, Steinacker J, Wabitsch M. Overweight prevention implemented by primary school teachers: a randomised controlled trial. Obesity facts. 2012 Mar 2;5(1):1-1.	Different target population
33	Briganti S, Zelaschi R, Ermetici F, Capitanio G, Romeo G, Cancellato A, Morricone L, Malavazos AE. The Italian E.A.T. Project: effectiveness of a multicomponent school-based health promotion study on measures of fatness and behavior in teenagers. Eating and weight disorders 2014; 19(3): 444-445.	Different target population
34	Buch-Andersen T, Eriksson F, Bloch P, Glümer C, Mikkelsen BE, Toft U. The Danish SoL Project: Effects of a Multi-Component Community-Based Health Promotion Intervention on Prevention of Overweight among 3–8-Year-Old Children. International Journal of Environmental Research and Public Health. 2021 Aug 9;18(16):8419.	Non-RCT
35	Bürgi F, Niederer I, Schindler C, Bodenmann P, Marques-Vidal P, Kriemler S, Puder JJ. Effect of a lifestyle intervention on adiposity and fitness in socially disadvantaged subgroups of preschoolers: a cluster-randomized trial (Ballabeina). Preventive medicine. 2012 May 1;54(5):335-40.	Different target population
36	Bustos N, Olivares S, Leyton B, Cano M, Albala C. Impact of a school-based intervention on nutritional education and physical activity in primary public schools in Chile (KIND) programme study protocol: cluster randomised controlled trial. BMC Public Health. 2016 Dec;16:1-1.	Study protocol
37	Cadenas-Sanchez C, Cabeza R, Idoate F, Osés M, Medrano M, Villanueva A, Arenaza L, Sanz A, Ortega FB, Ruiz JR, Labayen I. Effects of a family-based lifestyle intervention plus supervised exercise training on abdominal fat depots in children with overweight or obesity: a secondary analysis of a nonrandomized clinical trial. JAMA Network Open. 2022 Nov 1;5(11):e2243864-.	No central obesity outcome
38	Cao Z, Hua J, Zhang D, Thapa JR, Wang S. A cohort study assessing the sustainable long-term effectiveness of a childhood-obesity intervention in China. International journal of epidemiology. 2019 Feb 1;48(1):108-15.	No central obesity outcome
39	Cao Z, Wang S, Zheng W, Guo J, Qu S. Evaluation on the effectiveness of intervention comprehensive program on child obesity, using Generalized Estimating Equation. Zhonghua liu xing bing xue za zhi= Zhonghua liuxingbingxue zazhi. 2014 Jul 1;35(7):773-8.	No central obesity outcome
40	Cavero-Redondo I, Alvarez-Bueno C, Garrido-Miguel M, Martinez-Vizcaino V, Diez-Fernandez A, Fernandez-Infante A: Physical activity intervention program (movi-kids study) on preventing obesity in preschoolers	Reports not retrieved
41	Cezard G, Bansal N, Bhopal R, Pallan M, Gill P, Barrett T, Adab P. Adiposity and response to an obesity prevention intervention in Pakistani and Bangladeshi primary school boys and girls: a secondary analysis using the BEACHeS feasibility study. BMJ open. 2016 Feb 1;6(2):e007907.	Non-RCT
42	Chai LK, Collins CE, May C, Ashman A, Holder C, Brown LJ, Burrows TL. Feasibility and efficacy of a web-based family telehealth nutrition intervention to improve child weight status and dietary intake: a pilot randomised controlled trial. Journal of telemedicine and telecare. 2021 Apr;27(3):146-58.	Different target population

© 2025 Aychiluhm SB et al. *JAMA Network Open*.

43	Chaput JP, Leduc G, Boyer C, Belanger P, LeBlanc AG, Borghese MM, Tremblay MS. Objectively measured physical activity, sedentary time and sleep duration: independent and combined associations with adiposity in canadian children. Nutrition & diabetes. 2014 Jun;4(6):e117-.	Non-RCT
44	Chavarro JE, Peterson KE, Sobol AM, Wiecha JL, Gortmaker SL. Effects of a school-based obesity-prevention intervention on menarche (United States). Cancer Causes & Control. 2005 Dec;16(10):1245-52.	Different target population
45	Chen JL, Kwan M, Mac A, Chin NC, Liu K. iStart smart: a primary-care based and community partnered childhood obesity management program for Chinese-American children: feasibility study. Journal of immigrant and minority health. 2013 Dec;15:1125-8.	Non-RCT
46	Chen, R.; Ai, Z.; Yang, X.; Zhang, Y.; Yuan, X.: Effect of probiotics intake on obese children 2019	Reports not retrieved
47	Chen Y, Ma L, Ma Y, Wang H, Luo J, Zhang X, Luo C, Wang H, Zhao H, Pan D, Zhu Y. A national school-based health lifestyles interventions among Chinese children and adolescents against obesity: rationale, design and methodology of a randomized controlled trial in China. BMC public health. 2015 Dec;15:1-0.	Different target population
48	Christison AL, Evans TA, Bleess BB, Wang H, Aldag JC, Binns HJ. Exergaming for health: a randomized study of community-based exergaming curriculum in pediatric weight management. Games for health journal. 2016 Dec 1;5(6):413-21.	No central obesity outcome
49	Cloutier MM, Wiley J, Huedo-Medina T, Ohannessian CM, Grant A, Hernandez D, Gorin AA. Outcomes from a pediatric primary care weight management program: steps to growing up healthy. The Journal of pediatrics. 2015 Aug 1;167(2):372-7.	Different target population
50	Cohen TR, Hazell TJ, Vanstone CA, Rodd C, Weiler HA. A family-centered lifestyle intervention for obese six-to eight-year-old children: Results from a one-year randomized controlled trial conducted in Montreal, Canada. Canadian Journal of Public Health. 2016 Jul;107(4):e453-60.	Different target population
51	Comeras-Chueca C, Villalba-Heredia L, Perez-Lasierra JL, Marín-Puyalto J, Lozano-Berges G, Matute-Llorente Á, Vicente-Rodríguez G, Gonzalez-Aguero A, Casajús JA. Active video games improve muscular fitness and motor skills in children with overweight or obesity. International journal of environmental research and public health. 2022 Feb 24;19(5):2642.	No central obesity outcome
52	Costa CS, Rauber F, Leffa PS, Sangalli CN, Campagnolo PD, Vitolo MR. Ultra-processed food consumption and its effects on anthropometric and glucose profile: a longitudinal study during childhood. Nutrition, Metabolism and Cardiovascular Diseases. 2019 Feb 1;29(2):177-84.	Different target population
53	Cunha DB, Junior EV, Paravidino VB, Araújo MC, Mediano MF, Sgambato MR, de Souza BD, Marques ES, Baltar VT, de Oliveira AS, da Silva AC. Design of a school randomized trial for nudging students towards healthy diet and physical activity to prevent obesity: PAAPAS Nudge study protocol. Medicine. 2017 Dec 1;96(50):e8898.	Study protocol
54	Danielsson P, Kowalski J, Ekblom Ö, Marcus C. Response of severely obese children and adolescents to behavioral treatment. Archives of pediatrics & adolescent medicine. 2012 Dec 1;166(12):1103-8.	Non-RCT
55	Davis CL, Pollock NK, Waller JL, Allison JD, Dennis BA, Bassali R, Meléndez A, Boyle CA, Gower BA. Exercise dose and diabetes risk in overweight and obese children: a randomized controlled trial. Jama. 2012 Sep 19;308(11):1103-12.	Different target population
56	De Henauw S, Huybrechts I, De Bourdeaudhuij I, Bammann K, Barba G, Lissner L, Mårild S, Molnár D, Moreno LA, Pigeot I, Tornaritis M. Effects of a community-oriented obesity prevention programme on indicators of body fatness in preschool and primary school children. Main results from the IDEFICS study. obesity reviews. 2015 Dec;16:16-29.	Non-RCT
57	de Ruyter JC, Olthof MR, Seidell JC, Katan MB. A trial of sugar-free or sugar-sweetened beverages and body weight in children. New England Journal of Medicine. 2012 Oct 11;367(15):1397-406.	Different target population
58	Demir Acar M, Bayat M. The effect of diet-exercise trainings provided to overweight and obese teenagers through creative drama on their knowledge, attitude, and behaviors. Childhood Obesity. 2019 Feb 1;15(2):93-104.	Non-RCT

© 2025 Aychiluhm SB et al. *JAMA Network Open*.

59	Derwig M, Tiberg I, Björk J, Welander Tärneberg A, Hallström IK. A child-centered health dialogue for the prevention of obesity in child health services in Sweden–A randomized controlled trial including an economic evaluation. Obesity Science & Practice. 2022 Feb;8(1):77-90.	Different target population
60	Doyle-Baker PK, Venner AA, Lyon ME, Fung T. Impact of a combined diet and progressive exercise intervention for overweight and obese children: the BEHIP study. Applied Physiology, Nutrition, and Metabolism. 2011 Aug;36(4):515-25.	Different target population
61	Dreyhaupt J, Koch B, Wirt T, Schreiber A, Brandstetter S, Kesztyüs D, Wartha O, Kobel S, Kettner S, Prokopchuk D, Hundsdörfer V. Evaluation of a health promotion program in children: study protocol and design of the cluster-randomized Baden-Württemberg primary school study [DRKS-ID: DRKS00000494]. BMC Public Health. 2012 Dec;12:1-2.	Study protocol
62	Dreyhaupt J, Koch B, Wirt T, Schreiber A, Brandstetter S, Kesztyüs D, Wartha O, Kobel S, Kettner S, Prokopchuk D, Hundsdörfer V. Evaluation of a health promotion program in children: study protocol and design of the cluster-randomized Baden-Württemberg primary school study [DRKS-ID: DRKS00000494]. BMC Public Health. 2012 Dec;12:1-2.	Duplicated
63	Drummy C, Murtagh EM, McKee DP, Breslin G, Davison GW, Murphy MH. The effect of a classroom activity break on physical activity levels and adiposity in primary school children. Journal of paediatrics and child health. 2016 Jul;52(7):745-9.	Different target population
64	Fitzgibbon ML, Stolley MR, Schiffer LA, Braunschweig CL, Gomez SL, Van Horn L, Dyer AR. Hip-Hop to Health Jr. obesity prevention effectiveness trial: postintervention results. Obesity. 2011 May;19(5):994-1003.	Different target population
65	Ek A, Delisle Nyström C, Chirita-Emandi A, Tur JA, Nordin K, Bouzas C, Argelich E, Martínez JA, Frost G, Garcia-Perez I, Saez M. A randomized controlled trial for overweight and obesity in preschoolers: the More and Less Europe study-an intervention within the STOP project. BMC Public Health. 2019 Dec;19:1-3.	Study protocol
66	Ek A, Chamberlain KL, Ejderhamn J, Fisher PA, Marcus C, Chamberlain P, Nowicka P. The More and Less Study: a randomized controlled trial testing different approaches to treat obesity in preschoolers. BMC public health. 2015 Dec;15:1-7.	Study protocol
67	Elder JP, Crespo NC, Corder K, Ayala GX, Slymen DJ, Lopez NV, Moody JS, McKenzie TL. Childhood obesity prevention and control in city recreation centres and family homes: the MOVE/me M uevo P roject. Pediatric obesity. 2014 Jun;9(3):218-31.	Different target population
68	Elnaggar RK. A Randomized, Controlled Trial on the Effectiveness of Photobiomodulation Therapy and Non-Contact Selective-Field Radiofrequency on Abdominal Adiposity in Adolescents With Obesity. Lasers in Surgery and Medicine. 2020 Nov;52(9):873-81.	Different target population
69	Emmanouil CC, Pervanidou P, Charmandari E, Darviri C, Chrousos GP. The effectiveness of a health promotion and stress-management intervention program in a sample of obese children and adolescents. Hormones. 2018 Sep;17:405-13.	No central obesity outcome
70	Epstein LH, Paluch RA, Wrotniak BH, Daniel TO, Kilanowski C, Wilfley D, Finkelstein E. Cost-effectiveness of family-based group treatment for child and parental obesity. Childhood Obesity. 2014 Apr 1;10(2):114-21.	No central obesity outcome
71	Ezendam NP, Brug J, Oenema A. Evaluation of the Web-based computer-tailored FATaintPHAT intervention to promote energy balance among adolescents: results from a school cluster randomized trial. Archives of pediatrics & adolescent medicine. 2012 Mar 5;166(3):248-55.	Different target population
72	Fatahi S, Sayyari AA, Salehi M, Safa M, Sohouli M, Shidfar F, Santos HO. The effects of chitosan supplementation on anthropometric indicators of obesity, lipid and glycemic profiles, and appetite-regulated hormones in adolescents with overweight or obesity: A randomized, double-blind clinical trial. BMC pediatrics. 2022 Sep 5;22(1):527.	Different target population
73	Fernández-Ruiz VE, Solé-Agustí M, Armero-Barranco D, Cauli O. Weight loss and improvement of metabolic alterations in overweight and obese children through the I2AO2 family program: A randomized controlled clinical trial. Biological Research For Nursing. 2021 Jul;23(3):488-503.	Different target population

© 2025 Aychiluhm SB et al. *JAMA Network Open*.

74	Figueroa-Colon R, Franklin FA, Lee JY, Von Almen TK, Suskind RM. Feasibility of a clinic-based hypocaloric dietary intervention implemented in a school setting for obese children. Obesity Research. 1996 Sep;4(5):419-29.	Different target population
75	Flodmark CE, Ohlsson T, Rydén O, Sveger T. Prevention of progression to severe obesity in a group of obese schoolchildren treated with family therapy. Pediatrics. 1993 May 1;91(5):880-4.	No central obesity outcome
76	HEALTHY Study Group. A school-based intervention for diabetes risk reduction. New England Journal of Medicine. 2010 Jul 29;363(5):443-53.	Different target population
77	Foster GD, Sherman S, Borradaile KE, Grundy KM, Vander Veur SS, Nachmani J, Karpyn A, Kumanyika S, Shults J. A policy-based school intervention to prevent overweight and obesity. Pediatrics. 2008 Apr 1;121(4):e794-802.	Different target population
78	Friedrich RR, Caetano LC, Schiffner MD, Wagner MB, Schuch I. Design, randomization and methodology of the TriAtiva Program to reduce obesity in school children in Southern Brazil. BMC Public Health. 2015 Dec;15:1-8.	Study protocol
79	Gately PJ, King NA, Greatwood HC, Humphrey LC, Radley D, Cooke CB, Hill AJ. Does a high-protein diet improve weight loss in overweight and obese children?. Obesity. 2007 Jun;15(6):1527-34.	No central obesity outcome
80	Gatto NM, Martinez LC, Spruijt-Metz D, Davis JN. Retracted: LA sprouts randomized controlled nutrition and gardening program reduces obesity and metabolic risk in latino youth. Obesity. 2015 Jun;23(6):1244-51.	No central obesity outcome and retracted
81	Gesell SB, Bess KD, Barkin SL. Understanding the social networks that form within the context of an obesity prevention intervention. Journal of obesity. 2012;2012(1):749832.	Different target population
82	Ghayour-Mobarhan M, Sahebkar A, Vakili R, Safarian M, Nematy M, Lotfian E, Khorashadizadeh M, Tavallaie S, Dahri M, Ferns G. Investigation of the effect of high dairy diet on body mass index and body fat in overweight and obese children. The Indian Journal of Pediatrics. 2009 Nov;76:1145-50.	Non-RCT
83	Giralt M, Albaladejo R, Tarro L, Moriña D, Arija V, Solà R. A primary-school-based study to reduce prevalence of childhood obesity in Catalunya (Spain)-EDAL-Educació en alimentació: study protocol for a randomised controlled trial. Trials. 2011 Dec;12:1-5.	Study protocol
84	Goldfield GS, Epstein LH, Kilanowski CK, Paluch RA, Kogut-Bossler B. Cost-effectiveness of group and mixed family-based treatment for childhood obesity. International Journal of Obesity. 2001 Dec;25(12):1843-9.	Study protocol
85	Kilic Yildirim G, Dinleyici M, Vandenplas Y, Dinleyici EC. Effects of synbiotic supplementation on intestinal microbiota composition in children and adolescents with exogenous obesity:(Probesity-2 trial). Gut pathogens. 2023 Jul 21;15(1):36.	No central obesity outcome
86	Graham D, Appleton S, Rush E, McLennan S, Reed P, Simmons D. Increasing activity and improving nutrition through a schools-based programme: Project Energize. 1. Design, programme, randomisation and evaluation methodology. Public Health Nutrition. 2008 Oct;11(10):1076-84.	Study protocol
87	Grammer AC, Best JR, Fowler LA, Balantekin KN, Stein RI, Conlon RP, Saelens BE, Welch RR, Perri MG, Epstein LH, Wilfley DE. General and eating disorder psychopathology in relation to short-and long-term weight change in treatment-seeking children: a latent profile analysis. Annals of Behavioral Medicine. 2021 Jul 28;55(7):698-704.	No central obesity outcome
88	Grunseit AC, Taylor AJ, Hardy LL, King L. Composite measures quantify households' obesogenic potential and adolescents' risk behaviors. Pediatrics. 2011 Aug 1;128(2):e308-16.	Non-RCT
89	Grydeland M, Bjelland M, Anderssen SA, Klepp KI, Bergh IH, Andersen LF, Ommundsen Y, Lien N. Effects of a 20-month cluster randomised controlled school-based intervention trial on BMI of school-aged boys and girls: the HEIA study. British journal of sports medicine. 2014 May 1;48(9):768-73.	Different target population

© 2025 Aychiluhm SB et al. *JAMA Network Open*.

90	Gunawardena N, Kurotani K, Indrawansa S, Nonaka D, Mizoue T, Samarasinghe D. School-based intervention to enable school children to act as change agents on weight, physical activity and diet of their mothers: a cluster randomized controlled trial. International Journal of Behavioral Nutrition and Physical Activity. 2016 Dec;13:1-0.	Different target population
91	GÜNGÖRENLER C, ŞİMŞEK T. The Effect of Core Stabilization Exercises on Upper Extremity Motor Skills in Overweight and Obese Children: Quasi-Experimental Controlled Study. Türkiye Klinikleri Pediatri Dergisi. 2023;32(2).	Non-RCT
92	Habib-Mourad C, Ghandour LA, Maliha C, Dagher M, Kharroubi S, Hwalla N. Impact of a three-year obesity prevention study on healthy behaviors and BMI among Lebanese schoolchildren: Findings from ajyal salima program. Nutrients. 2020 Sep 3;12(9):2687.	No central obesity outcome
93	Habib-Mourad C, Ghandour LA, Moore HJ, Nabhani-Zeidan M, Adetayo K, Hwalla N, Summerbell C. Promoting healthy eating and physical activity among school children: findings from Health-E-PALS, the first pilot intervention from Lebanon. BMC public health. 2014 Dec;14:1-1.	Different target population
94	Hadley TS, Cave TL, Derraik JG, Hofman PL, Anderson YC. Associations between changes in caregiver's and child's weight status in a community-based obesity intervention programme. International Journal of Obesity. 2022 Jul;46(7):1406-9.	No central obesity outcome
95	Hajihashemi P, Azadbakht L, Hashemipor M, Kelishadi R, Esmaillzadeh A. Whole-grain intake favorably affects markers of systemic inflammation in obese children: a randomized controlled crossover clinical trial. Molecular nutrition & food research. 2014 Jun;58(6):1301-8.	Duplicate
96	Hakanen M, Lagström H, Kaitosaari T, Niinikoski H, Näntö-Salonen K, Jokinen E, Sillanmäki L, Viikari J, Rönnemaa T, Simell O. Development of overweight in an atherosclerosis prevention trial starting in early childhood. The STRIP study. International journal of obesity. 2006 Apr;30(4):618-26.	Different target population
97	Hawthorne A, Shaibi G, Gance-Cleveland B, McFall S. Grand Canyon Trekkers: school-based lunchtime walking program. The Journal of School Nursing. 2011 Feb;27(1):43-50.	Non-RCT
98	Herrera L, Strong D, Knight R, Rhee K: Effect of probiotics and dietary changes on adiposity in children	Reports not retrieved
99	Hollar D, Lopez-Mitnik G, Hollar L, Messiah S. 1442 Elementary School-Based Obesity Prevention Intervention Effect on Waist Circumference Among Multiethnic 6–13 Year Olds. Archives of Disease in Childhood. 2012 Oct 1;97(Suppl 2):A410-.	Non-RCT
100	Hollis JL, Sutherland R, Campbell L, Morgan PJ, Lubans DR, Nathan N, Wolfenden L, Okely AD, Davies L, Williams A, Cohen KE. Effects of a 'school-based'physical activity intervention on adiposity in adolescents from economically disadvantaged communities: secondary outcomes of the 'Physical Activity 4 Everyone'RCT. International Journal of Obesity. 2016 Oct;40(10):1486-93.	Different target population
101	Hopkins LC, Fristad M, Goodway JD, Eneli I, Holloman C, Kennel JA, Melnyk B, Gunther C. Camp NERF: methods of a theory-based nutrition education recreation and fitness program aimed at preventing unhealthy weight gain in underserved elementary children during summer months. BMC Public Health. 2016 Dec;16:1-2.	Different target population
102	Howie EK, McVeigh JA, Abbott RA, Olds TS, Straker LM. Multiple components of fitness improved among overweight and obese adolescents following a community-based lifestyle intervention. Journal of Sports Sciences. 2016 Aug 17;34(16):1581-7.	Different target population
103	Hsu CH, Hwang KC, Chao CL, Lin JG, Kao ST, Chou P. Effects of electroacupuncture in reducing weight and waist circumference in obese women: a randomized crossover trial. International journal of obesity. 2005 Nov;29(11):1379-84.	Different target population
104	Hu Y, He JR, Liu FH, Li WD, Lu JH, Xing YF, Lin SF, Liu X, Bartington S, Feng Q, Xia HM. Effectiveness of a kindergarten-based intervention for preventing childhood obesity. Pediatrics. 2017 Dec 1;140(6).	Non-RCT
105	Huang T, Larsen KT, Jepsen JR, Møller NC, Thorsen AK, Mortensen EL, Andersen LB. Effects of an obesity intervention program on cognitive function in children: A randomized controlled trial. Obesity. 2015 Oct;23(10):2101-8.	No central obesity outcome

© 2025 Aychiluhm SB et al. *JAMA Network Open*.

106	Hughes AR, Stewart L, Chapple J, McColl JH, Donaldson MD, Kelnar CJ, Zabihollah M, Ahmed F, Reilly JJ. Randomized, controlled trial of a best-practice individualized behavioral program for treatment of childhood overweight: Scottish Childhood Overweight Treatment Trial (SCOTT). Pediatrics. 2008 Mar 1;121(3):e539-46.	No central obesity outcome
107	Hume C, Singh A, Brug J, van Mechelen W, Chinapaw M. Dose-response associations between screen time and overweight among youth. International journal of pediatric obesity. 2009 Mar;4(1):61-4.	Non-RCT
108	Hung SH, Hwang SL, Su MJ, Lue SH, Hsu CY, Chen HL, Chen HS. An evaluation of a weight-loss program incorporating E-learning for obese junior high school students. Telemedicine and e-Health. 2008 Oct 1;14(8):783-92.	Non-RCT
109	Yang HJ, Kang JH, Kim OH, Choi M, Oh M, Nam J, Sung E. Interventions for preventing childhood obesity with smartphones and wearable device: a protocol for a non-randomized controlled trial. International journal of environmental research and public health. 2017 Feb;14(2):184.	Study protocol
110	James J, Thomas P, Kerr D. Preventing childhood obesity: two year follow-up results from the Christchurch obesity prevention programme in schools (CHOPPS). Bmj. 2007 Oct 11;335(7623):762.	Different target population
111	Janicke DM, Lim CS, Perri MG, Bobroff LB, Mathews AE, Brumback BA, Dumont-Driscoll M, Silverstein JH. The extension family lifestyle intervention project (E-FLIP for Kids): Design and methods. Contemporary clinical trials. 2011 Jan 1;32(1):50-8.	Study protocol
112	Jansen W, Raat H, Zwanenburg EJ, Reuvers I, van Walsem R, Brug J. A school-based intervention to reduce overweight and inactivity in children aged 6–12 years: study design of a randomized controlled trial. BMC Public Health. 2008 Dec;8:1-9.	Study protocol
113	Jia E, Zhu H, Geng H, Liu R, Wo X, Zeng Y, Ma W, Yao X, Zhan Z, Zhang J. The effects of aerobic exercise on body composition in overweight and obese patients with gout: a randomized, open-labeled, controlled trial. Trials. 2022 Sep 5;23(1):745.	Different target population
114	Jiang J, Xia X, Greiner T, Wu G, Lian G, Rosenqvist U. The effects of a 3-year obesity intervention in schoolchildren in Beijing. Child: care, health and development. 2007 Sep;33(5):641-6.	Different target population
115	Espinoza Silva JM, Latorre Román PÁ, Cabrera Linares JC, Párraga Montilla JA, Martínez Salazar C. Effects of a high intensity interval training (HIIT) program on anthropomorphic and cardiometabolic variables in school children with overweight and obesity. Children. 2023 Feb 7;10(2):317.	Different target population
116	Kafatos A, Manios Y, Moschandreas J. Health and nutrition education in primary schools of Crete: follow-up changes in body mass index and overweight status. European journal of clinical nutrition. 2005 Sep;59(9):1090-2.	No central obesity outcome
117	Kain J, Concha F, Moreno L, Leyton B. School-based obesity prevention intervention in Chilean children: effective in controlling, but not reducing obesity. Journal of obesity. 2014;2014(1):618293.	No central obesity outcome
118	Kain J, Uauy R, Leyton B, Cerda R, Olivares S, Vio F. Effectiveness of a dietary and physical activity intervention to prevent obesity in school age children. Revista medica de Chile. 2008 Jan 1;136(1):22-30.	No central obesity outcome
119	Kalavainen MP, Korppi MO, Nuutinen OM. Clinical efficacy of group-based treatment for childhood obesity compared with routinely given individual counseling. International journal of obesity. 2007 Oct;31(10):1500-8.	No central obesity outcome
120	Kelishadi R, Hashemipour M, Sarrafzadegan N, Mohammadifard N, Alikhasy H, Beizaei M, Sajjadi F, Poursafa P, Amin Z, Ghatreh-Samani S, Khavarian N. Effects of a lifestyle modification trial among phenotypically obese metabolically normal and phenotypically obese metabolically abnormal adolescents in comparison with phenotypically normal metabolically obese adolescents. Maternal & Child Nutrition. 2010 Jul;6(3):275-86.	Non-RCT
121	Keller A, Klossek A, Gausche R, Hoepffner W, Kiess W, Keller E. Selective primary obesity prevention in children. Deutsche Medizinische Wochenschrift (1946). 2008 Dec 17;134(1-2):13-8.	Different target population

© 2025 Aychiluhm SB et al. *JAMA Network Open*.

122	Kesztyüs D, Lauer R, Kesztyüs T, Kilian R, Steinacker JM, “Join the Healthy Boat” Study Group. Costs and effects of a state-wide health promotion program in primary schools in Germany–the Baden-Württemberg Study: A cluster-randomized, controlled trial. PLoS One. 2017 Feb 21;12(2):e0172332.	Different target population
123	Kesztyüs D, Schreiber A, Wirt T, Wiedom M, Dreyhaupt J, Brandstetter S, Koch B, Wartha O, Muche R, Wabitsch M, Kilian R. Economic evaluation of URMEL-ICE, a school-based overweight prevention programme comprising metabolism, exercise and lifestyle intervention in children. The European Journal of Health Economics. 2013 Apr;14:185-95.	Different target population
124	Khan NA, Raine LB, Drollette ES, Scudder MR, Pontifex MB, Castelli DM, Donovan SM, Evans EM, Hillman CH. Impact of the FITKids physical activity intervention on adiposity in prepubertal children. Pediatrics. 2014 Apr 1;133(4):e875-83.	Different target population
125	Kilic Yildirim G, Dinleyici M, Vandenplas Y, Dinleyici EC. Effects of multispecies synbiotic supplementation on anthropometric measurements, glucose and lipid parameters in children with exogenous obesity: A randomized, double blind, placebo-controlled clinical trial (Probesity-2 trial). Frontiers in Nutrition. 2022 Jul 1;9:898037.	No central obesity outcome
126	Knop C, Singer V, Uysal Y, Schaefer A, Wolters B, Reinehr T. Extremely obese children respond better than extremely obese adolescents to lifestyle interventions. Pediatric obesity. 2015 Feb;10(1):7-14.	No central obesity outcome
127	Kocken PL, Scholten AM, Westhoff E, De Kok BP, Taal EM, Goldbohm RA. Effects of a theory-based education program to prevent overweightness in primary school children. Nutrients. 2016 Jan 4;8(1):12.	Different target population
128	Kokkvoll A, Grimsgaard S, Ødegaard R, Flægstad T, Njølstad I. Single versus multiple-family intervention in childhood overweight—Finnmark Activity School: a randomised trial. Archives of disease in childhood. 2014 Mar 1;99(3):225-31.	No central obesity outcome
129	Kong AS, Sussman AL, Yahne C, Skipper BJ, Burge MR, Davis SM. School-based health center intervention improves body mass index in overweight and obese adolescents. Journal of Obesity. 2013;2013(1):575016.	Non-RCT
130	Kong XJ, Liu K, Zhuang P, Tian R, Liu S, Clairmont C, Lin X, Sherman H, Zhu J, Wang Y, Fong M. The Effects of Limosilactobacillus reuteri LR-99 Supplementation on Body Mass Index, Social Communication, Fine Motor Function, and Gut Microbiome Composition in Individuals with Prader–Willi Syndrome: a Randomized Double-Blinded Placebo-Controlled Trial. Probiotics and Antimicrobial Proteins. 2021 Dec;13:1508-20.	No central obesity outcome
131	Koo HC, Poh BK, Abd Talib R. The GReat-Child™ Trial: A quasi-experimental intervention on whole grains with healthy balanced diet to manage childhood obesity in Kuala Lumpur, Malaysia. Nutrients. 2018 Jan 30;10(2):156.	Non-RCT
132	Koo HC, Poh BK, Talib RA. The GReat-child TrialTM: A quasi-experimental dietary intervention among overweight and obese children. Nutrients. 2020 Sep 29;12(10):2972.	Non-RCT
133	Kriemler S, Zahner L, Schindler C, Meyer U, Hartmann T, Hebestreit H, Brunner-La Rocca HP, Van Mechelen W, Puder JJ. Effect of school based physical activity programme (KISS) on fitness and adiposity in primary schoolchildren: cluster randomised controlled trial. Bmj. 2010 Feb 24;340.	Different target population
134	Labayen I, Medrano M, Arenaza L, Maiz E, Oses M, Martinez-Vizcaino V, Ruiz JR, Ortega FB. Effects of exercise in addition to a family-based lifestyle intervention program on hepatic fat in children with overweight. Diabetes Care. 2020 Feb 1;43(2):306-13.	Non-RCT
135	Lambourne K, Washburn RA, Gibson C, Sullivan DK, Goetz J, Lee R, Smith BK, Mayo MS, Donnelly JE. Weight management by phone conference call: a comparison with a traditional face-to-face clinic. Rationale and design for a randomized equivalence trial. Contemporary clinical trials. 2012 Sep 1;33(5):1044-55.	Different target population
136	Landau Z, Abiri S, Lebenthal Y, Jakubowicz D, Mor N, Lerner-Geva L, Boaz M, Wainstein J, Bar-Dayan Y. Lifestyle intervention program benefits children with overweigh compared to children with obesity. Obesity research & clinical practice. 2018 Jan 1;12(1):85-92.	Non-RCT

© 2025 Aychiluhm SB et al. *JAMA Network Open*.

137	Lanigan J, Collins S, Birbara T, Kokoreli M, Singhal A. The TrimTots programme for prevention and treatment of obesity in preschool children: evidence from two randomised controlled trials. The Lancet. 2013 Nov 29;382:S58.	Different target population
138	Lee ST, Wong JE, Nik Shanita S, Ismail MN, Deurenberg P, Poh BK. Daily physical activity and screen time, but not other sedentary activities, are associated with measures of obesity during childhood. International journal of environmental research and public health. 2015 Jan;12(1):146-61.	Non-RCT
139	Lejeune MP, Kovacs EM, Westerterp-Plantenga MS. Additional protein intake limits weight regain after weight loss in humans. British Journal of Nutrition. 2005 Feb;93(2):281-9.	Different target population
140	Leme AC, Lubans DR, Guerra PH, Dewar D, Toassa EC, Philippi ST. Preventing obesity among Brazilian adolescent girls: six-month outcomes of the Healthy Habits, Healthy Girls–Brazil school-based randomized controlled trial. Preventive medicine. 2016 May 1;86:77-83.	Duplicate
141	Li B, Liu WJ, Adab P, Pallan M, Hemming K, Frew E, Lin R, Martin J, Liu W, Cheng KK. Cluster-randomised controlled trial to assess the effectiveness and cost-effectiveness of an obesity prevention programme for Chinese primary school-aged children: the CHIRPY DRAGON study protocol. BMJ open. 2017 Nov 1;7(11):e018415.	Study protocol
142	Li B, Pallan M, Liu WJ, Hemming K, Frew E, Lin R, Liu W, Martin J, Zanganeh M, Hurley K, Cheng KK. The CHIRPY DRAGON intervention in preventing obesity in Chinese primary-school--aged children: A cluster-randomised controlled trial. PLoS Medicine. 2019 Nov 26;16(11):e1002971.	Different target population
143	Li XH, Lin S, Guo H, Huang Y, Wu L, Zhang Z, Ma J, Wang HJ. Effectiveness of a school-based physical activity intervention on obesity in school children: a nonrandomized controlled trial. BMC public health. 2014 Dec;14:1-2.	Non-RCT
144	Yan-Ping LI, Xiao-Qi HU, Schouten EG, Ai-Ling LI, Song-Ming DU, Lin-Zhong LI, Zhao-Hui CU, Dong WA, Frans JK, Frank BH, Guan-Sheng MA. Report on childhood obesity in China (8): effects and sustainability of physical activity intervention on body composition of Chinese youth. Biomedical and Environmental Sciences. 2010 Jun 1;23(3):180-7.	Different target population
145	Liu Z, Gao P, Gao AY, Lin Y, Feng XX, Zhang F, Xu LQ, Niu WY, Fang H, Zhou S, Li WH. Effectiveness of a multifaceted intervention for prevention of obesity in primary school children in China: a cluster randomized clinical trial. JAMA pediatrics. 2022 Jan 1;176(1):e214375-.	Different target population
146	Liu Z, Li Q, Maddison R, Ni Mhurchu C, Jiang Y, Wei DM, Cheng L, Cheng Y, Wang D, Wang HJ. A school-based comprehensive intervention for childhood obesity in China: a cluster randomized controlled trial. Childhood Obesity. 2019 Feb 1;15(2):105-15.	Different target population
147	Liu Z, Wu Y, Niu WY, Feng X, Lin Y, Gao A, Zhang F, Fang H, Pei GA, Li HJ, Wang H. A school-based, multi-faceted health promotion programme to prevent obesity among children: protocol of a cluster-randomised controlled trial (the DECIDE-Children study). BMJ open. 2019 Nov 1;9(11):e027902.	Different target population
148	Llargués E, Recasens A, Franco R, Nadal A, Vila M, Pérez MJ, Recasens I, Salvador G, Serra J, Roure E, Castell C. Medium-term evaluation of an educational intervention on dietary and physical exercise habits in schoolchildren: the Avall 2 study. Endocrinología y Nutrición (English Edition). 2012 May 1;59(5):288-95.	Non-RCT
149	Llargués E, Recasens MA, Manresa JM, Jensen BB, Franco R, Nadal A, Vila M, Recasens I, Pérez MJ, Castell C. Four-year outcomes of an educational intervention in healthy habits in schoolchildren: the Avall 3 Trial. The European Journal of Public Health. 2017 Feb 1;27(1):42-7.	Different target population
150	Lloyd JJ, Wyatt KM, Creanor S. Behavioural and weight status outcomes from an exploratory trial of the Healthy Lifestyles Programme (HeLP): a novel school-based obesity prevention programme. BMJ open. 2012 Jan 1;2(3):e000390.	Duplicate

© 2025 Aychiluhm SB et al. *JAMA Network Open*.

151	Lloyd J, Creanor S, Price L, Abraham C, Dean S, Green C, Hillsdon M, Pearson V, Taylor RS, Tomlinson R, Logan S. Trial baseline characteristics of a cluster randomised controlled trial of a school-located obesity prevention programme; the Healthy Lifestyles Programme (HeLP) trial. BMC Public Health. 2017 Dec;17:1-4.	Different target population
152	Lopes J, Jove Cesar D, Oliveira AL, Silva AF, Biasi BL, Santos VB, Silva RP. Effect of a supervised exercise program on overweight and obese adolescents: a quasi-experimental study. European Heart Journal. 2021 Oct 1;42(Supplement_1):ehab724-3014.	Non-RCT
153	Lu, Y.; He, G. A quasi-experimental study of a health educational intervention targeting parents of overweight and obese pre-school children in Shanghai, China	Non-RCT
154	Lubans DR, Morgan PJ, Okely AD, Dewar D, Collins CE, Batterham M, Callister R, Plotnikoff RC. Preventing obesity among adolescent girls: one-year outcomes of the nutrition and enjoyable activity for teen girls (NEAT Girls) cluster randomized controlled trial. Archives of pediatrics & adolescent medicine. 2012 Sep 1;166(9):821-7.	Different target population
155	Lubans DR, Sheaman C, Callister R. Exercise adherence and intervention effects of two school-based resistance training programs for adolescents. Preventive medicine. 2010 Jan 1;50(1-2):56-62.	Different target population
156	Luque V, Feliu A, Escribano J, Ferré N, Flores G, Monné R, Gutiérrez-Marín D, Guillen N, Muñoz-Hernando J, Zaragoza-Jordana M, Gispert-Llauradó M. The Obemat2. 0 study: a clinical trial of a motivational intervention for childhood obesity treatment. Nutrients. 2019 Feb 16;11(2):419.	Different target population
157	Luque V, Feliu A, Escribano J, Ferré N, Flores G, Monné R, Gutiérrez-Marín D, Guillen N, Muñoz-Hernando J, Zaragoza-Jordana M, Gispert-Llauradó M. The Obemat2. 0 study: a clinical trial of a motivational intervention for childhood obesity treatment. Nutrients. 2019 Feb 16;11(2):419.	Duplicate
158	Mabli J, Bleeker M, Fox MK, Jean-Louis B, Fox M. Randomized controlled trial of healthy Harlem's get fit program: an after-school intervention for childhood overweight and obesity in the Harlem Children's zone. Childhood Obesity. 2020 Oct 1;16(7):479-87.	No central obesity outcome
159	Macieira L, Saraiva J, da Conceição dos Santos L. Short-and Medium-Term Impact of a Structured Medical Intervention in Adolescents with Overweight, Obesity, or Increased Waist Circumference. Obesity Facts. 2021 Oct 5;14(6):622-32	Non-RCT
160	Maddison R, Foley L, Ni Mhurchu C, Jull A, Jiang Y, Prapavessis H, Rodgers A, Vander Hoorn S, Hohepa M, Schaaf D. Feasibility, design and conduct of a pragmatic randomized controlled trial to reduce overweight and obesity in children: The electronic games to aid motivation to exercise (eGAME) study. BMC public health. 2009 Dec;9:1-9.	Non-RCT
161	Maddison R, Mhurchu CN, Foley L, Epstein L, Jiang Y, Tsai M, Dewes O, Heke I. Screen-time Weight-loss Intervention Targeting Children at Home (SWITCH): a randomized controlled trial study protocol. BMC Public Health. 2011 Dec;11:1-9.	Study protocol
162	Magnusson KT, Hrafnkelsson H, Sigurgeirsson I, Johannsson E, Sveinsson T. Limited effects of a 2-year school-based physical activity intervention on body composition and cardiorespiratory fitness in 7-year-old children. Health education research. 2012 Jun 1;27(3):484-94.	Different target population
163	Cohen TR, Mak IL, Loiselle SE, Kasvis P, Hazell TJ, Vanstone CA, Rodd C, Weiler HA. Changes in Adiposity without Impacting Bone Health in 9-to 12-Year-Old Children with Overweight and Obesity after a One-Year Family-Centered Lifestyle Behaviour Intervention. Childhood Obesity. 2023 Jan 1;19(1):46-56.	Different target population
164	Malarvizhi D, Dinesh AM. Effectiveness of Lumbar Flexors and Extensors Muscle Strengthening Exercises on Waist-Hip Ratio in Overweight Adolescents. EXECUTIVE EDITOR. 2018 Sep;9(9):21.	Non-RCT
165	Marcus C, Nyberg G, Nordenfelt A, Karpmyr M, Kowalski J, Ekelund U. A 4-year, cluster-randomized, controlled childhood obesity prevention study: STOPP. International journal of obesity. 2009 Apr;33(4):408-17.	Different target population

© 2025 Aychiluhm SB et al. *JAMA Network Open*.

166	Marsigliante S, Gómez-López M, Muscella A. Effects on children's physical and mental well-being of a physical-activity-based school intervention program: a randomized study. International Journal of Environmental Research and Public Health. 2023 Jan 20;20(3):1927.	Different target population
167	Martínez Vizcaíno V, Salcedo Aguilar F, Franquelo Gutiérrez R, Solera Martínez M, Sánchez López M, Serrano Martínez S, Lopez Garcia E, Rodriguez Artalejo F. Assessment of an after-school physical activity program to prevent obesity among 9-to 10-year-old children: a cluster randomized trial. International journal of obesity. 2008 Jan;32(1):12-22.	No central obesity outcome
168	Martínez-Vizcaino V, Mota J, Solera-Martínez M, Notario-Pacheco B, Arias-Palencia N, García-Prieto JC, González-García A, Álvarez-Bueno C, Sánchez-López M, MOVI-KIDS group. Rationale and methods of a randomised cross-over cluster trial to assess the effectiveness of MOVI-KIDS on preventing obesity in pre-schoolers. BMC Public Health. 2015 Dec;15:1-9.	No central obesity outcome
169	Matvienko O, Ahrabi-Fard I. The effects of a 4-week after-school program on motor skills and fitness of kindergarten and first-grade students. American Journal of Health Promotion. 2010 May;24(5):299-303.	Non-RCT
170	McCallum Z, Wake M, Gerner B, Baur LA, Gibbons K, Gold L, Gunn J, Harris C, Naughton G, Riess C, Sanci L. Outcome data from the LEAP (Live, Eat and Play) trial: a randomized controlled trial of a primary care intervention for childhood overweight/mild obesity. International journal of obesity. 2007 Apr;31(4):630-6.	No central obesity outcome
171	Mcmurray RG, Harrell JS, Bangdiwala SI, Bradley CB, Deng S, Levine A. A school-based intervention can reduce body fat and blood pressure in young adolescents. Journal of Adolescent Health. 2002 Aug 1;31(2):125-32.	No central obesity outcome
172	Meng L, Xu H, Liu A, van Raaij J, Bemelmans W, Hu X, Zhang Q, Du S, Fang H, Ma J, Xu G. The costs and cost-effectiveness of a school-based comprehensive intervention study on childhood obesity in China. PloS one. 2013 Oct 18;8(10):e77971.	Different target population
173	Mihas C, Mariolis A, Manios Y, Naska A, Arapaki A, Mariolis-Sapsakos T, Tountas Y. Evaluation of a nutrition intervention in adolescents of an urban area in Greece: short-and long-term effects of the VYRONAS study. Public health nutrition. 2010 May;13(5):712-9.	Different target population
174	Miller J, Shoemaker AH, Jennifer AM, Gottschalk M, Yuan G, Malhotra S, Scimia C, Roth CL. Impact of setmelanotide treatment on weight-and body composition-related outcomes in pediatric and adult patients with hypothalamic obesity. InEndocrine Abstracts 2023 May 2 (Vol. 90). Bioscientifica.	Report not retrieved
175	Mirza NM, Palmer MG, Sinclair KB, McCarter R, He J, Ebbeling CB, Ludwig DS, Yanovski JA. Effects of a low glycemic load or a low-fat dietary intervention on body weight in obese Hispanic American children and adolescents: a randomized controlled trial. The American journal of clinical nutrition. 2013 Feb 1;97(2):276-85.	Different target population
176	Kassim MS, Manaf MR, Nor NS, Ambak R. Effects of lifestyle intervention towards obesity and blood pressure among housewives in Klang Valley: a quasi-experimental study. The Malaysian journal of medical sciences: MJMS. 2017 Dec;24(6):83.	Non-RCT
177	Morell-Azanza L, Ojeda-Rodríguez A, Azcona-SanJulián MC, Zalba G, Marti A. Associations of telomere length with anthropometric and glucose changes after a lifestyle intervention in abdominal obese children. Nutrition, Metabolism and Cardiovascular Diseases. 2020 Apr 12;30(4):694-700.	Non-RCT
178	Moya Martínez P, Sánchez López M, López Bastida J, Escribano Sotos F, Notario Pacheco B, Salcedo Aguilar F, Martínez Vizcaíno V. Coste-efectividad de un programa de actividad física de tiempo libre para prevenir el sobrepeso y la obesidad en niños de 9-10 años. Gaceta Sanitaria. 2011;25:198-204.	Not in English
179	Müller UM, Walther C, Adams V, Mende M, Adam J, Fikenzer K, Machalica KC, Erbs S, Linke A, Schuler G. Long term impact of one daily unit of physical exercise at school on cardiovascular risk factors in school children. European journal of preventive cardiology. 2016 Sep 1;23(13):1444-52.	Different target population
180	Nemet D, Barzilay-Teeni N, Eliakim A. Treatment of childhood obesity in obese families. Journal of Pediatric Endocrinology and Metabolism. 2008 May;21(5):461-8.	No central obesity outcome

© 2025 Aychiluhm SB et al. *JAMA Network Open*.

181	Nemet D, Oren S, Pantanowitz M, Eliakim A. Effects of a multidisciplinary childhood obesity treatment intervention on adipocytokines, inflammatory and growth mediators. Hormone research in paediatrics. 2013 Jul 1;79(6):325-32.	Non-RCT
182	Nicolucci AC, Hume MP, Martínez I, Mayengbam S, Walter J, Reimer RA. Prebiotics reduce body fat and alter intestinal microbiota in children who are overweight or with obesity. Gastroenterology. 2017 Sep 1;153(3):711-22.	No central obesity outcome
183	Niederer I, Bürgi F, Ebenegger V, Marques-Vidal P, Schindler C, Nydegger A, Kriemler S, Puder JJ. Effects of a lifestyle intervention on adiposity and fitness in overweight or low fit preschoolers (Ballabeina). Obesity. 2013 Mar;21(3):E287-93.	Different target population
184	Niederer I, Kriemler S, Zahner L, Bürgi F, Ebenegger V, Hartmann T, Meyer U, Schindler C, Nydegger A, Marques-Vidal P, Puder JJ. Influence of a lifestyle intervention in preschool children on physiological and psychological parameters (Ballabeina): study design of a cluster randomized controlled trial. BMC Public Health. 2009 Dec;9:1-1.	Study protocol
185	Suparyatmo JB, Prayitno A. The Effect of Nutrition Education on Body Mass Index, Waist Circumference, Mid-upper Arm Circumference and Blood Pressure in Obese Adolescents. Electronic Journal of General Medicine. 2020 Sep 1;17(5).	Non-RCT
186	Nowicka P, Höglund P, Pietrobelli A, Lissau I, Flodmark CE. Family Weight School treatment: 1-year results in obese adolescents. International Journal of Pediatric Obesity. 2008 Jan 1;3(3):141-7.	Different target population
187	Nowicka P, Lanke J, Pietrobelli A, Apitzsch E, Flodmark CE. Sports camp with six months of support from a local sports club as a treatment for childhood obesity. Scandinavian journal of public health. 2009 Nov;37(8):793-800.	Different target population
188	Nurul-Fadhilah A, Teo PS, Huybrechts I, Foo LH (2013) Infrequent Breakfast Consumption Is Associated with Higher Body Adiposity and Abdominal Obesity in Malaysian School-Aged Adolescents. PLoS ONE 8(3): e59297. https://doi.org/10.1371/journal.pone.0059297	Non-RCT
189	Ochoa-Avilés A, Verstraeten R, Huybregts L, Andrade S, Van Camp J, Donoso S, Ramírez PL, Lachat C, Maes L, Kolsteren P. A school-based intervention improved dietary intake outcomes and reduced waist circumference in adolescents: a cluster randomized controlled trial. Nutrition journal. 2017 Dec;16:1-2.	Different target population
190	Ochoa-Avilés A, Verstraeten R, Huybregts L, Andrade S, Van Camp J, Donoso S, Ramírez PL, Lachat C, Maes L, Kolsteren P. A school-based intervention improved dietary intake outcomes and reduced waist circumference in adolescents: a cluster randomized controlled trial. Nutrition journal. 2017 Dec;16:1-2.	Duplicated
191	A. Page, S. Munasinghe, S. Marjanovic, H. Jani, E. Conroy, L. Freebairn, on behalf of The Australian Prevention Partnership Centre. The Western Sydney Adolescent Health Study: A pilot study of behavioural factors associated with overweight and obesity. Translational Health Research Institute, Western Sydney University: Campbelltown.	Non-RCT
192	Olvera N, Scherer R, McLeod J, Graham M, Knox B, Hall K, Butte NF, Bush JA, Smith DW, Bloom J. BOUNCE: an exploratory healthy lifestyle summer intervention for girls. American Journal of Health Behavior. 2010 Mar 1;34(2):144-55.	Non-RCT
193	Owens S, Gutin B, Allison J, Riggs SH, Ferguson M, Litaker MA, Thompson WI. Effect of physical training on total and visceral fat in obese children. Medicine and science in sports and exercise. 1999 Jan 1;31(1):143-8.	Different target population
194	Parra-Medina D. Weight outcomes of Latino adults and children participating in the Y living program, a family-focused lifestyle intervention, San Antonio, 2012–2013. Preventing Chronic Disease. 2015;12.	Non-RCT
195	Parra-Medina D, Mojica C, Liang Y, Ouyang Y, Ramos AI, Gomez I. Promoting weight maintenance among overweight and obese Hispanic children in a rural practice. Childhood Obesity. 2015 Aug 1;11(4):355-63.	Non-RCT
196	Peña S, Carranza M, Cuadrado C, Parra DC, Villalobos Dintrans P, Castillo C, Cortinez-O'Ryan A, Espinoza P, Müller V, Rivera C, Genovesi R. Effectiveness of a gamification strategy to prevent childhood obesity in schools: a cluster controlled trial. Obesity. 2021 Nov;29(11):1825-34.	Different target population

© 2025 Aychiluhm SB et al. *JAMA Network Open*.

197	Peralta LR, Jones RA, Okely AD. Promoting healthy lifestyles among adolescent boys: the Fitness Improvement and Lifestyle Awareness Program RCT. Preventive medicine. 2009 Jun 1;48(6):537-42.	Different target population
198	Peterson KE, Fox MK. Addressing the epidemic of childhood obesity through school-based interventions: what has been done and where do we go from here?. Journal of Law, Medicine & Ethics. 2007 Apr;35(1):113-30.	Non-RCT
199	Pippi R, Mascherini G, Izzicupo P, Bini V, Fanelli CG. Effects of a mixed exercise program on overweight and obese children and adolescents: A pilot, uncontrolled study. International Journal of Environmental Research and Public Health. 2022 Jul 28;19(15):9258.	Non-RCT
200	Patriota PF, Filgueiras AR, de Almeida VB, Alexmovitz GA, da Silva CE, de Carvalho VF, Carvalho N, de Albuquerque MP, Domene SM, do Prado WL, Torres GE. Effectiveness of a 16-month multi-component and environmental school-based intervention for recovery of poor income overweight/obese children and adolescents: study protocol of the health multipliers program. BMC public health. 2017 Dec;17:1-3.	Study protocol
201	Ponnambalam S, Palanisamy S, Singaravelu R, Janardhanan HA. Effectiveness of after-school physical activity intervention on body mass index and waist circumference/height ratio among overweight adolescents in selected Schools at Puducherry, India: A randomized controlled trial. Indian Journal of Community Medicine. 2022 Jan 1;47(1):72-5.	Different target population
202	Puder JJ, Marques-Vidal P, Schindler C, Zahner L, Niederer I, Bürgi F, Ebenegger V, Nydegger A, Kriemler S. Effect of multidimensional lifestyle intervention on fitness and adiposity in predominantly migrant preschool children (Ballabeina): cluster randomised controlled trial. Bmj. 2011 Oct 13;343.	Different target population
203	Quattrin T, Roemmich JN, Paluch R, Yu J, Epstein LH, Ecker MA. Efficacy of family-based weight control program for preschool children in primary care. Pediatrics. 2012 Oct 1;130(4):660-6.	Different target population
204	Quattrin T, Roemmich JN, Paluch R, Yu J, Epstein LH, Ecker MA. Treatment outcomes of overweight children and parents in the medical home. Pediatrics. 2014 Aug 1;134(2):290-7.	Different target population
205	Raducha D, Ratajczak J, Jackowski T, Horodnicka-Józwa A, Szmit-Domagalska J, Walczak M, Petriczko E. Effects of 12-Month Interdisciplinary Interventions in 8-and 9-Year-Old Children with Excess Body Weight. International Journal of Environmental Research and Public Health. 2022 Nov 29;19(23):15899.	Non- RCT
206	Recasens MA, Xicola-Coromina E, Manresa JM, Ullmo PA, Jensen BB, Franco R, Suarez A, Nadal A, Vila M, Recasens I, Pérez MJ. Impact of school-based nutrition and physical activity intervention on body mass index eight years after cessation of randomized controlled trial (AVall study). Clinical nutrition. 2019 Dec 1;38(6):2592-8.	Different target population
207	Reinehr T, Bucksch J, Müller A, Finne E, Kolip P. 7-Year follow-up of a lifestyle intervention in overweight children: Comparison to an untreated control group. Clinical Nutrition. 2018 Oct 1;37(5):1558-62.	No central obesity outcome
208	Reinehr, T., Temmesfeld, M., Kersting, M. *et al.* Four-year follow-up of children and adolescents participating in an obesity intervention program. *Int J Obes* **31**, 1074–1077 (2007). https://doi.org/10.1038/sj.ijo.0803637.	Non- RCT
209	Reinehr T, Kleber M, de Sousa G, Andler W. Leptin concentrations are a predictor of overweight reduction in a lifestyle intervention. International journal of pediatric obesity. 2009 Jan 1;4(4):215-23.	No central obesity outcome
210	Reznik M, Wylie-Rosett J, Kim M, Ozuah PO. A classroom-based physical activity intervention for urban kindergarten and first-grade students: a feasibility study. Child Obes. 2015 Jun;11(3):314-24. doi: 10.1089/chi.2014.0090. Epub 2015 Mar 6. PMID: 25747719; PMCID: PMC4485880.	Non- RCT
211	Rizvi JZ, Kumar P, Kulkarni MM, Kamath A. Outcome of structured health education intervention for obesity-risk reduction among junior high school students: Stratified cluster randomized controlled trial (RCT) in South India. Journal of Education and Health Promotion. 2022 Jan 1;11(1):400.	Different target population

© 2025 Aychiluhm SB et al. *JAMA Network Open*.

212	Robinson TN. Reducing children's television viewing to prevent obesity: a randomized controlled trial. Jama. 1999 Oct 27;282(16):1561-7.	Different target population
213	Robinson TN, Matheson D, Desai M, Wilson DM, Weintraub DL, Haskell WL, McClain A, McClure S, Banda JA, Sanders LM, Haydel KF. Family, community and clinic collaboration to treat overweight and obese children: Stanford GOALS—a randomized controlled trial of a three-year, multi-component, multi-level, multi-setting intervention. Contemporary clinical trials. 2013 Nov 1;36(2):421-35.	Study protocol
214	Robinson TN, Matheson D, Wilson DM, Weintraub DL, Banda JA, McClain A, Sanders LM, Haskell WL, Haydel KF, Kapphahn KI, Pratt C. A community-based, multi-level, multi-setting, multi-component intervention to reduce weight gain among low socioeconomic status Latinx children with overweight or obesity: The Stanford GOALS randomised controlled trial. The Lancet Diabetes & Endocrinology. 2021 Jun 1;9(6):336-49.	No central obesity outcome
215	Rodrigues L, Oliveira M. ANTHROPOMETRIC, CLINICAL AND INFLAMMATORY PROFILE OF CHILDREN AND ADOLESCENTS WITH WEIGHT EXCESS BEFORE AND AFTER NUTRITIONAL INTERVENTION WITH OATMEAL. InANNALS OF NUTRITION AND METABOLISM 2017 Jan 1 (Vol. 71, pp. 591-591). ALLSCHWILERSTRASSE 10, CH-4009 BASEL, SWITZERLAND: KARGER.	Reports not retrieved
216	Rogovik AL, Goldman RD. Pharmacologic treatment of pediatric obesity. Canadian Family Physician. 2011 Feb;57(2):195.	No central obesity outcome
217	Rosenstock S, Ingalls A, Cuddy RF, Neault N, Littlepage S, Cohoe L, Nelson L, Shephard-Yazzie K, Yazzie S, Alikhani A, Reid R. Effect of a home-visiting intervention to reduce early childhood obesity among Native American children: a randomized clinical trial. JAMA pediatrics. 2021 Feb 1;175(2):133-42.	Different target population
218	Sacher PM, Kolotourou M, Chadwick PM, Cole TJ, Lawson MS, Lucas A, Singhal A. Randomized controlled trial of the MEND program: a family-based community intervention for childhood obesity. Obesity. 2010 Feb;18(S1):S62-8.	Duplicate
219	Sacher PM, Kolotourou M, Chadwick PM, Cole TJ, Lawson MS, Lucas A, Singhal A. Randomized controlled trial of the MEND program: a family-based community intervention for childhood obesity. Obesity. 2010 Feb;18(S1):S62-8.	Duplicate
220	Sadeghi, B., Kaiser, L.L., Hanbury, M.M. *et al.* A three-year multifaceted intervention to prevent obesity in children of Mexican-heritage. *BMC Public Health* **19**, 582 (2019). https://doi.org/10.1186/s12889-019-6897-8	Non- RCT
221	Sahota P, Rudolf MC, Dixey R, Hill AJ, Barth JH, Cade J. Randomised controlled trial of primary school based intervention to reduce risk factors for obesity. Bmj. 2001 Nov 3;323(7320):1029.	Different target population
222	Santos RG, Durksen A, Rabbani R, Chanoine JP, Miln AL, Mayer T, McGavock JM. Effectiveness of peer-based healthy living lesson plans on anthropometric measures and physical activity in elementary school students: a cluster randomized trial. JAMA pediatrics. 2014 Apr 1;168(4):330-7.	Different target population
223	Schaefer A, Winkel K, Finne E, Kolip P, Reinehr T. An effective lifestyle intervention in overweight children: one-year follow-up after the randomized controlled trial on “Obeldicks light”. Clinical Nutrition. 2011 Oct 1;30(5):629-33.	Different target population
224	Scherr RE, Linnell JD, Dharmar M, Beccarelli LM, Bergman JJ, Briggs M, Brian KM, Feenstra G, Hillhouse JC, Keen CL, Ontai LL. A multicomponent, school-based intervention, the shaping healthy choices program, improves nutrition-related outcomes. Journal of nutrition education and behavior. 2017 May 1;49(5):368-79.	Different target population
225	Seral-Cortes M, Sabroso-Lasa S, Bailo-Aysa A, Gonzalez-Gross M, Molnár D, Censi L, Molina-Hidalgo C, Gottrand F, Henauw SD, Manios Y, Mavrogianni C. Mediterranean diet, screen-time-based sedentary behavior and their interaction effect on adiposity in European adolescents: the HELENA study. Nutrients. 2021 Jan 30;13(2):474.	Non- RCT
226	Sgambato MR, Cunha DB, Souza BS, Henriques VT, Rodrigues RR, Rêgo AL, Pereira RA, Yokoo EM, Sichieri R. Effectiveness of school–home intervention for adolescent obesity prevention: parallel school randomised study. British Journal of Nutrition. 2019 Nov;122(9):1073-80.	Different target population

© 2025 Aychiluhm SB et al. *JAMA Network Open*.

227	Shahriarzadeh F, Kelishadi R, Fatehizadeh M, Hassanzadeh A, Askari G. The effect of motivational interviewing and healthy diet on anthropometric indices and blood pressure in overweight and obese school children. Journal of Isfahan Medical School. 2017 May 22;35(426):412-21.	Not English
228	Shoujiang W. Analysis of the impact of different exercise methods on the physical health of obese adolescents. Revista Brasileira de Medicina do Esporte. 2022 Aug 15;29:e2022_0172.	Non- RCT
229	Shrewsbury VA, Nguyen B, O'Connor J, Steinbeck KS, Lee A, Hill AJ, Shah S, Kohn MR, Torvaldsen S, Baur LA. Short-term outcomes of community-based adolescent weight management: The Loozit® Study. BMC pediatrics. 2011 Dec;11:1-0.	Non- RCT
230	Siegrist M, Hanssen H, Lammel C, Haller B, Halle M. A cluster randomised school-based lifestyle intervention programme for the prevention of childhood obesity and related early cardiovascular disease (JuvenTUM 3). BMC Public Health. 2011 Dec;11:1-0.	Study protocol
231	Sigmund E, Sigmundová D. Longitudinal 2-year follow-up on the effect of a non-randomised school-based physical activity intervention on reducing overweight and obesity of Czech children aged 10–12 years. International Journal of Environmental Research and Public Health. 2013 Aug;10(8):3667-83.	Non- RCT
232	Simons M, Chinapaw MJ, van de Bovenkamp M, de Boer MR, Seidell JC, Brug J, de Vet E. Active video games as a tool to prevent excessive weight gain in adolescents: rationale, design and methods of a randomized controlled trial. BMC public health. 2014 Dec;14:1-3.	Study protocol
233	Singh AS, Paw MJ, Brug J, van Mechelen W. Dutch obesity intervention in teenagers: effectiveness of a school-based program on body composition and behavior. Archives of pediatrics & adolescent medicine. 2009 Apr 6;163(4):309-17.	Different target population
234	Singh AS, Paw MJ, Brug J, van Mechelen W. Short-term effects of school-based weight gain prevention among adolescents. Archives of pediatrics & adolescent medicine. 2007 Jun 1;161(6):565-71.	Different target population
235	Solbrig L, Whalley B, Kavanagh DJ, May J, Parkin T, Jones R, Andrade J. Functional imagery training versus motivational interviewing for weight loss: a randomised controlled trial of brief individual interventions for overweight and obesity. International Journal of Obesity. 2019 Apr;43(4):883-94.	Different target population
236	Sothern MS, von Almen TK, Schumacher HD, Suskind RM, Blecker U. A multidisciplinary approach to the treatment of childhood obesity. Del Med J. 1999 Jun;71(6):255-61. PMID: 10432772.	Reports not retrieved
237	Sutherland R, Reeves P, Campbell E, Lubans DR, Morgan PJ, Nathan N, Wolfenden L, Okely AD, Gillham K, Davies L, Wiggers J. Cost effectiveness of a multi-component school-based physical activity intervention targeting adolescents: the ‘Physical Activity 4 Everyone’cluster randomized trial. international journal of behavioral nutrition and physical activity. 2016 Dec;13:1-4.	No central obesity outcome
238	Takacs H, Martos E, Kovacs VA. Effects of a practice-focused nutrition intervention in Hungarian adolescents. Annali dell'Istituto Superiore di Sanità. 2020 Mar 25;56(1):99-106.	Different target population
239	Tan-Ting AM, Llido L. Outcome of a hospital based multidisciplinary weight loss program in obese Filipino children. Nutrition. 2011 Jan 1;27(1):50-4.	Non- RCT
240	Taylor RW, McAuley KA, Barbezat W, Strong A, Williams SM, Mann JI. APPLE Project: 2-y findings of a community-based obesity prevention program in primary school–age children. The American journal of clinical nutrition. 2007 Sep 1;86(3):735-42.	Different target population
241	Tian X, Fu J, Tian J, Yang Y, Liang W, Fan W, Zhao R. The Efficacy of Brief School-Based Exercise Programs in Improving Pubertal Bone Mass and Physical Fitness: A Randomized Controlled Trial. International Journal of Environmental Research and Public Health. 2021 Sep 13;18(18):9648.	No central obesity outcome

© 2025 Aychiluhm SB et al. *JAMA Network Open*.

242	Tognon, G.; Lauria, F.; Siani, A.; Russo, P.; Hebestreit, A.; Foraita, R.; Veidebaum, T.; Tornaritis, M.; Molnar, D.; De Henauw, S.; Moreno, L. A.; Krogh, V.; Lissner, L. .The FTO variant rs9939609 is associated with 5-year change in BMI, waist circumference and percent fat mass in children from 8 European countries	Reports not retrieved
243	Toulabi T, Nikoo MK, Amini F, Nazari H, Mardani M. The influence of a behavior modification interventional program on body mass index in obese adolescents. Journal of the Formosan Medical Association. 2012 Mar 1;111(3):153-9.	Different target population
244	Trinh A, Campbell M, Ukoumunne OC, Gerner B, Wake M. Physical activity and 3-year BMI change in overweight and obese children. Pediatrics. 2013 Feb 1;131(2):e470-7.	Non- RCT
245	Trost SG, Sundal D, Foster GD, Lent MR, Vojta D. Effects of a pediatric weight management program with and without active video games: a randomized trial. JAMA pediatrics. 2014 May 1;168(5):407-13.	Different target population
246	van Grieken A, Veldhuis L, Renders CM, Borsboom GJ, van der Wouden JC, Hirasing RA, Raat H. Population-based childhood overweight prevention: outcomes of the 'Be active, eat right'study. PloS one. 2013 May 31;8(5):e65376.	Different target population
247	van Schayck CP, Willeboordse M, Oosterhof M, Bartelink N, van Assema P, Kremers S, Winkens B, Savelberg H, Jansen M, Joore M, Vreugdenhil AC. Effects and costs of The Healthy Primary School of the Future. Nederlands Tijdschrift Voor Geneeskunde. 2021 Jan 28;165:D4979-.	Non- RCT
248	VanEvery H, Pacheco LS, Sun E, Allison MA, Gao X. The impact of avocado intake on anthropometric measures among Hispanic/Latino children and adolescents: A cluster randomized controlled trial. Clinical Nutrition ESPEN. 2023 Aug 1;56:94-103.	Different target population
249	Vanhelst J, Deken V, Boulic G, Raffin S, Duhamel A, Romon M. Trends in prevalence of childhood overweight and obesity in a community-based programme: The VIF Programme. Pediatric Obesity. 2021 Jul;16(7):e12761.	Non- RCT
250	Verbeken S, Braet C, Naets T, Houben K, Boendermaker W. Computer training of attention and inhibition for youngsters with obesity: A pilot study. Appetite. 2018 Apr 1;123:439-47.	Non- RCT
251	Vignolo M, Rossi F, Bardazza G, Pistorio A, Parodi A, Spigno S, Torrisi C, Gremmo M, Veneselli E, Aicardi G. Five-year follow-up of a cognitive-behavioural lifestyle multidisciplinary programme for childhood obesity outpatient treatment. European journal of clinical nutrition. 2008 Sep;62(9):1047-57.	Non- RCT
252	Wake M, Baur LA, Gerner B, Gibbons K, Gold L, Gunn J, Levickis P, McCallum Z, Naughton G, Sanci L, Ukoumunne OC. Outcomes and costs of primary care surveillance and intervention for overweight or obese children: the LEAP 2 randomised controlled trial. Bmj. 2009 Sep 3;339.	Different target population
253	Wake M, Price A, Clifford S, Ukoumunne OC, Hiscock H. Does an intervention that improves infant sleep also improve overweight at age 6? Follow-up of a randomised trial. Archives of disease in childhood. 2011 Jun 1;96(6):526-32.	Different target population
254	Wang X, Liu J, Gao D, Li Y, Ma Q, Chen L, Chen M, Ma T, Ma Y, Zhang Y, Yang J. Effectiveness of national multicentric school-based health lifestyles intervention among chinese children and adolescents on knowledge, belief, and practice toward obesity at individual, family and schools' levels. Frontiers in Pediatrics. 2022 Aug 18;10:917376.	Different target population
255	Wang Z, Xu F, Ye Q, Tse LA, Xue H, Tan Z, Leslie E, Owen N, Wang Y. Childhood obesity prevention through a community-based cluster randomized controlled physical activity intervention among schools in china: the health legacy project of the 2nd world summer youth olympic Games (YOG-Obesity study). International Journal of Obesity. 2018 Apr;42(4):625-33.	Different target population
256	Warren JM, Henry CJ, Lightowler HJ, Bradshaw SM, Perwaiz S. Evaluation of a pilot school programme aimed at the prevention of obesity in children. Health promotion international. 2003 Dec 1;18(4):287-96.	Non- RCT

© 2025 Aychiluhm SB et al. *JAMA Network Open*.

257	Waters E, Gibbs L, Tadic M, Ukoumunne OC, Magarey A, Okely AD, de Silva A, Armit C, Green J, O'Connor T, Johnson B. Cluster randomised trial of a school-community child health promotion and obesity prevention intervention: findings from the evaluation of fun 'n healthy in Moreland!. BMC public health. 2018 Dec;18:1-6.	Different target population
258	Waters, E., Gibbs, L., Tadic, M. *et al.* Cluster randomised trial of a school-community child health promotion and obesity prevention intervention: findings from the evaluation of *fun 'n healthy in Moreland!* . *BMC Public Health* **18**, 92 (2018). https://doi.org/10.1186/s12889-017-4625-9	Duplicated
259	Weigel C, Kokocinski K, Lederer P, Dötsch J, Rascher W, Knerr I. Childhood obesity: concept, feasibility, and interim results of a local group-based, long-term treatment program. Journal of nutrition education and behavior. 2008 Nov 1;40(6):369-73.	Different target population
260	Wen LM, Taki S, Xu H, Phongsavan P, Rissel C, Hayes A, Baur LA. Effectiveness and co-benefits of a telephone-based intervention in reducing obesity risk of children aged 2–4 years: findings from a pragmatic randomised controlled trial during the COVID-19 pandemic in Australia. The Lancet Global Health. 2023 Mar 1;11:S13.	Different target population
261	Willeboordse M, Bartelink NH, van Assema P, Kremers SP, Savelberg HH, Hahnraths MT, Vonk L, Oosterhoff M, Van Schayck CP, Winkens B, Jansen MW. Battling the obesity epidemic with a school-based intervention: Long-term effects of a quasi-experimental study. Plos one. 2022 Sep 27;17(9):e0272291.	Non- RCT
262	Willeboordse M, Jansen MW, Van den Heijkant SN, Simons A, Winkens B, de Groot RH, Bartelink N, Kremers SP, Van Assema P, Savelberg HH, De Neubourg E. The Healthy Primary School of the Future: study protocol of a quasi-experimental study. BMC Public Health. 2016 Dec;16:1-3.	Study protocol
263	Wyatt KM, Lloyd JJ, Abraham C, Creanor S, Dean S, Densham E, Daurge W, Green C, Hillsdon M, Pearson V, Taylor RS. The Healthy Lifestyles Programme (HeLP), a novel school-based intervention to prevent obesity in school children: study protocol for a randomised controlled trial. Trials. 2013 Dec;14:1-2.	Study protocol
264	Xu F, Wang X, Ware RS, Tse LA, Wang Z, Hong X, Chan EY, Li J, Wang Y. A school-based comprehensive lifestyle intervention among Chinese kids against Obesity (CLICK-Obesity) in Nanjing City, China: the baseline data. Asia Pacific journal of clinical nutrition. 2014;23(1):48.	Different target population
265	Xu F, Ware RS, Tse LA, Wang Z, Hong X, Song A, Li J, Wang Y. A school-based comprehensive lifestyle intervention among chinese kids against obesity (CLICK-Obesity): rationale, design and methodology of a randomized controlled trial in Nanjing city, China. BMC public health. 2012 Dec;12:1-8.	Study protocol
266	Xu H, Li Y, Zhang Q, Hu X, Liu A, Du S, Li T, Guo H, Li Y, Xu G, Liu W. Comprehensive school-based intervention to control overweight and obesity in China: a cluster randomized controlled trial. Asia Pacific Journal of Clinical Nutrition. 2017 Jan;26(6):1139-51.	Different target population
267	Yang JJ, Xing HJ, Wang SJ, Xiao HL, Li M, Li Q. Effects of acupuncture combined with dietary adjustments and aerobic exercise on body weight, body mass index and serum leptin level in simple obesity patients. Zhen ci yan jiu= Acupuncture Research. 2010 Dec 1;35(6):453-7.	Different target population
268	Yang Y, Kang B, Lee EY, Yang HK, Kim HS, Lim SY, Lee JH, Lee SS, Suh BK, Yoon KH. Effect of an obesity prevention program focused on motivating environments in childhood: a school-based prospective study. International Journal of Obesity. 2017 Jul;41(7):1027-34.	Non- RCT
269	Yu S, Gao Y, Wang A, Sun Y, Wang J, Kwok HH, Wu S, Lam CK, Tao ED, Jiao JJ, Fong SS. Effectiveness of an adapted physical activity intervention for weight management in adolescents with intellectual disability: A randomized controlled trial. Pediatric obesity. 2022 May;17(5):e12882.	Different target population
270	Zehsaz F, Farhangi N, Ghahramani M. The response of circulating omentin-1 concentration to 16-week exercise training in male children with obesity. The Physician and sportsmedicine. 2016 Oct 1;44(4):355-61.	Non- RCT

© 2025 Aychiluhm SB et al. *JAMA Network Open*.

271	Zoorob R, Buchowski MS, Beech BM, Canedo JR, Chandrasekhar R, Akohoue S, Hull PC. Healthy families study: Design of a childhood obesity prevention trial for Hispanic families. Contemporary Clinical Trials. 2013 Jul 1;35(2):108-21.	Study protocol
272	Bäcklund C, Sundelin G, Larsson C. Effect of a 1-year lifestyle intervention on physical activity in overweight and obese children. Advances in Physiotherapy. 2011 Sep 1;13(3):87-96.	Non-RCT
273	Boutelle KN, Rhee KE, Manzano MA, Bernard RS, Strong DR, Eichen DM, Anderson CC, Marcus BH, Akshoomoff N, Crow SJ. Design of the FRESH-DOSE study: A randomized controlled noninferiority trial evaluating a guided self-help family-based treatment program for children with overweight or obesity. Contemporary clinical trials. 2023 Jan 1;124:106996.	Study protocol
274	Singhal N, Misra A. A school-based intervention for diabetes risk reduction. The New England journal of medicine. 2010 Oct 28;363(18):1769-70.	Different target population
275	Smith JJ, Morgan PJ, Plotnikoff RC, Dally KA, Salmon J, Okely AD, Finn TL, Lubans DR. Smart-phone obesity prevention trial for adolescent boys in low-income communities: the ATLAS RCT. Pediatrics. 2014 Sep 1;134(3):e723-31.	Different target population
276	Warnakulasuriya, L. S.; Fernando, M. A. M.; Adikaram, A. V. N.; Thawfeek, A. R. M.; Anurasiri, W. M. L.; Silva, K. D. R.; Sirasa, M. S. F.; Samaranayake, D.; Wickramasinghe, V. P. Metformin in the management of childhood obesity in Sri Lankan setting	Report not retrieved
277	Laroche HH, Andino J, O'Shea AM, Engebretsen B, Rice S, DeJear Jr M, Nicholson C, Yeh HW, Snetselaar L. Family-Based Motivational Interviewing and Resource Mobilization to Prevent Obesity: Living Well Together Trial. Journal of Nutrition Education and Behavior. 2024 Jun 19.	No central obesity outcome
278	Rhee KE, Corbett T, Patel S, Eichen DM, Strong DR, Anderson C, Marcus B, Boutelle KN. A randomized controlled trial examining general parenting training and family-based behavioral treatment for childhood obesity: The ReFRESH study design. Contemporary Clinical Trials. 2024 Jul 1;142:107562.	Study protocol
279	Boutelle KN, Manzano MA, Pasquale EK, Bernard RS, Strong DR, Rhee KE, Eichen DM, Engel S, Miller A, Peterson CB. Design of the FRESH-teen study: A randomized controlled trial evaluating an adapted emotion regulation weight loss program for adolescents with overweight or obesity and their parent. Contemporary Clinical Trials. 2024 Oct 1;145:107640.	No central obesity outcome
280	Wang D, Katalambula LK, Modest AR, Ismail A, Malero A, Bray D, Cinq-Mars H, Tinkasimile A, Sando MM, Vuai S, Fawzi WW. Meals, Education, and Gardens for In-School Adolescents: A Cluster Randomized Trial of an Adolescent Nutrition Intervention Package in Tanzania. Journal of Adolescent Health. 2024 Apr 8.	No central obesity outcome
281	van der Baan-Slootweg O, Benninga MA, Beelen A, van der Palen J, Tamminga-Smeulders C, Tijssen JG, van Aalderen WM. Inpatient treatment of children and adolescents with severe obesity in the Netherlands: a randomized clinical trial. JAMA pediatrics. 2014 Sep 1;168(9):807-14.	Different target population
282	Patterson E, Nyberg G, Norman Å, Schäfer Elinder L. Universal healthy school start intervention reduced the body mass index of young children with obesity. Acta Paediatrica. 2024 Feb 21.	No central obesity outcome

© 2025 Aychiluhm SB et al. *JAMA Network Open*.

eTable 4: multiple meta-regression model

Covariates	Estimate	SE	p-value	95%CI
Intercept	128.7024	285.5655	0.6583	-476.6694 734.0741
Year	-0.0654	0.1427	0.6528	-0.3678 0.2370
Economic Region_Lower middle income	-0.1436	1.3958	0.9194	-3.1026 2.8155
Economic Region_Upper middle income	-0.7584	1.5593	0.6333	-4.0640 2.5472
Intervention_TypeD,PA&B	0.7384	1.3198	0.5836	-2.0594 3.5363
Intervention_TypeD&PA	0.1595	1.7332	0.9278	-3.5146 3.8336
Intervention_TypeDiet	1.8049	2.8414	0.5343	-4.2186 7.8285
Intervention_TypeDietary Supplement	2.0639	2.0400	0.3267	-2.2607 6.3884
Intervention_TypeMotivational	2.4861	2.9166	0.4066	-3.6968 8.6690
Intervention_TypePA	0.1463	1.7290	0.9336	-3.5190 3.8115
Intervention_TypePharmacotherapy	-3.6597	1.9193	0.0747	-7.7284 0.4089
Intervention_Setting_Healthcare Facility	-0.6697	1.7370	0.7049	-4.3521 3.0126
Intervention_Setting_Home	0.4771	2.1696	0.8287	-4.1223 5.0765
Intervention_Setting_School	2.4055	3.3547	0.4837	-4.7062 9.5172
Intervention_Duration_above 12	0.9993	1.4462	0.4995	-2.0666 4.0652
Design_RCT	2.2370	1.4704	0.1477	-0.8802 5.3541

*=Statistically significant at 95% confidence interval. The maximum likelihood (ML) estimation method was used to estimate the residual between-study variance ($τ^2$ = 1.2639, SE = 0.3231), with τ (the square root of $τ^2$) estimated at 1.1242. The degree of heterogeneity across studies was substantial, as indicated by the high I^2 value (97.44%), suggesting that 97.44% of the total variability in effect sizes was due to real differences between studies rather than chance. The model explained 51.90% of the observed heterogeneity (R^2), meaning that nearly half of the variability was accounted for by the included moderators. The overall test of moderators was not statistically significant ($F(17,16)$ = 0.8432, p = 0.6354), indicating that the included predictors did not significantly explain the variation in effect sizes across studies.

© 2025 Aychiluhm SB et al. *JAMA Network Open*.

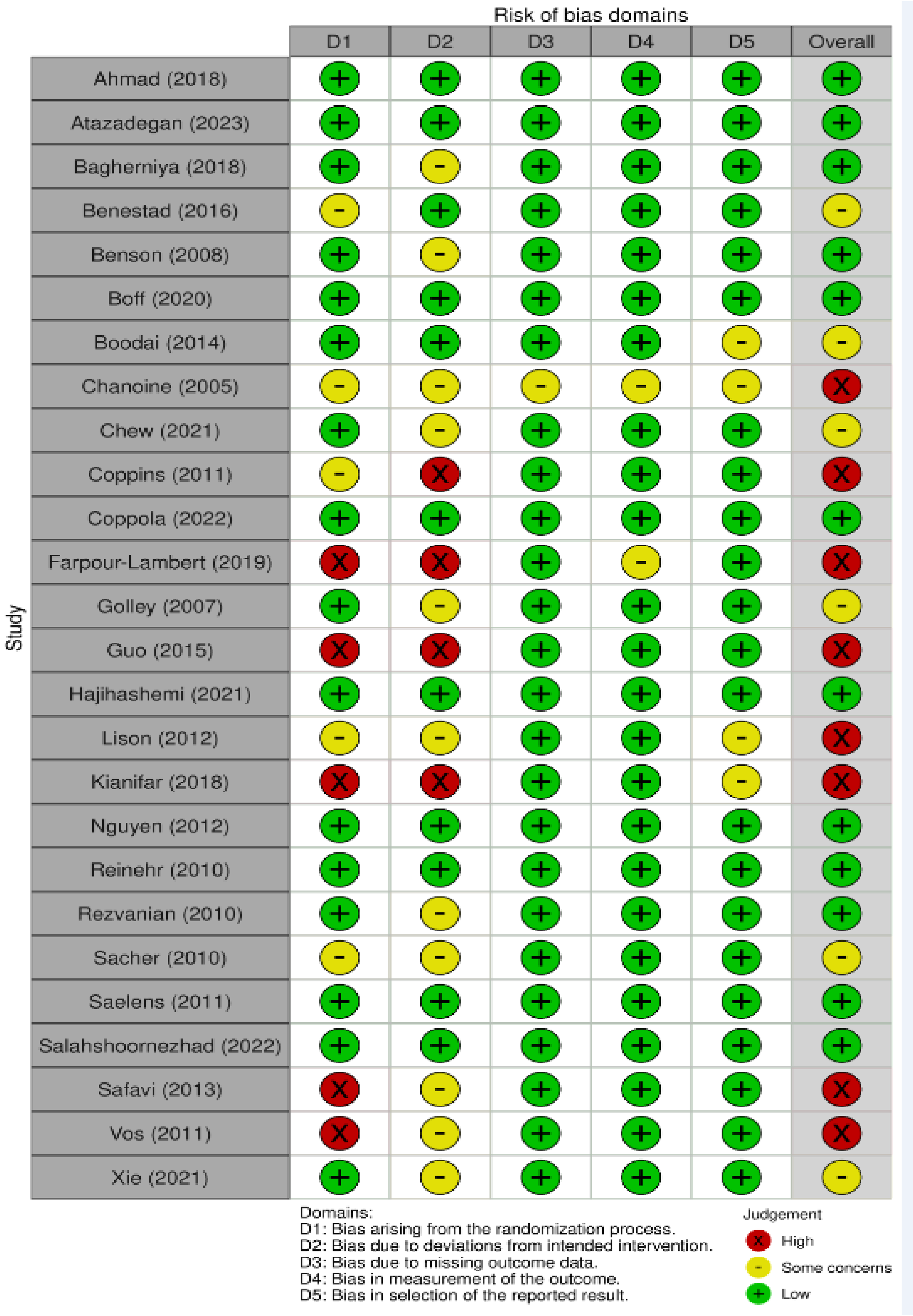

eFigure 1. Risk of bias traffic light plot for selected individual RCT articles.

© 2025 Aychiluhm SB et al. *JAMA Network Open*.

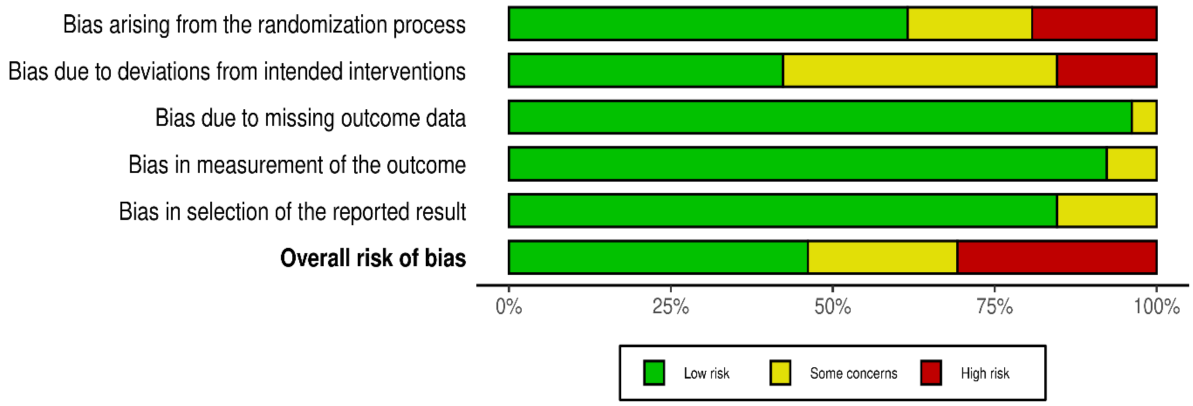

eFigure 2. Risk of bias summary plot for selected individual RCT articles.

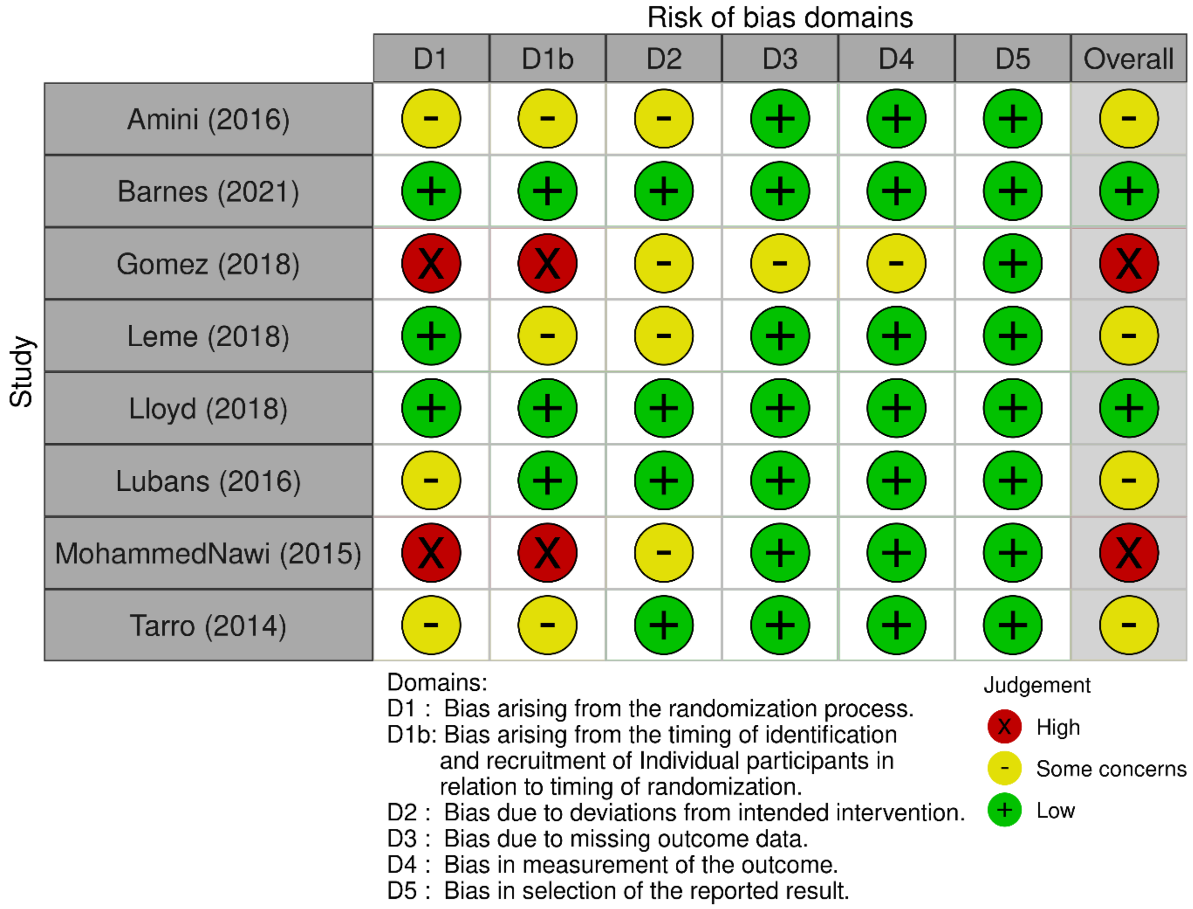

eFigure 3. Risk of bias traffic light plot for selected cluster RCT articles

© 2025 Aychiluhm SB et al. *JAMA Network Open*.

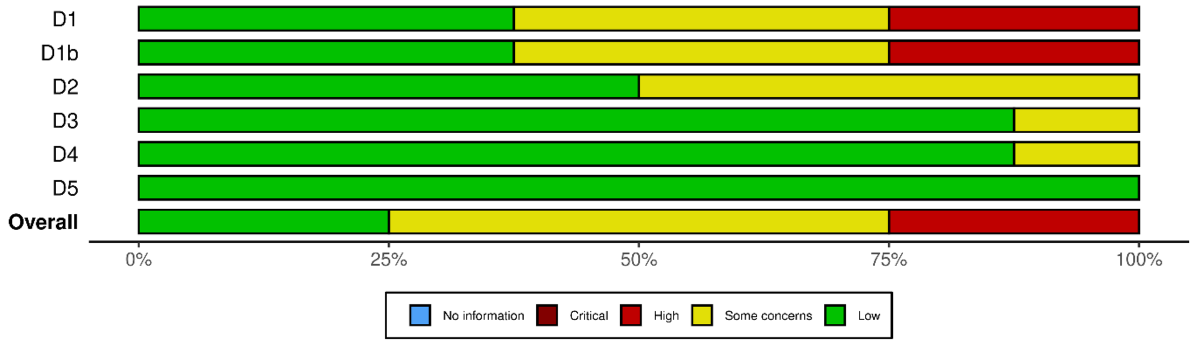

eFigure 4. Risk of bias summary plot for selected cluster RCT articles

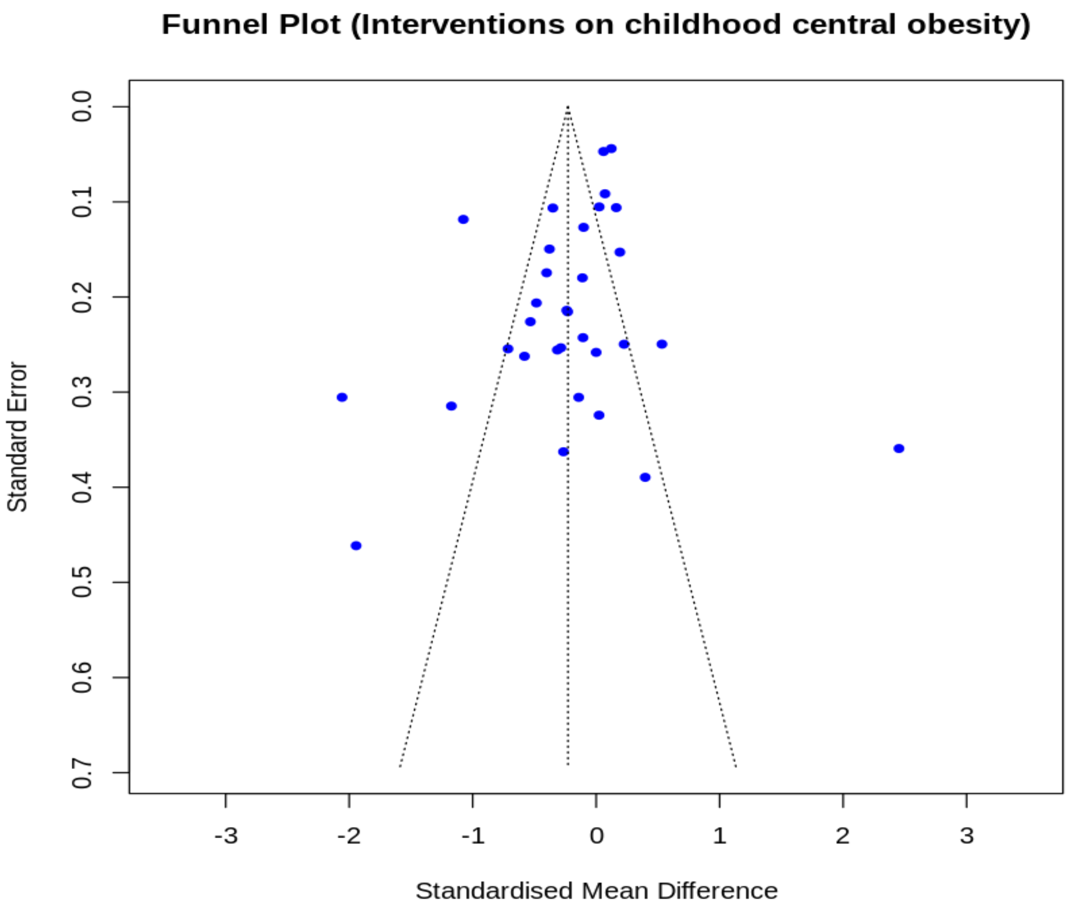

eFigure 5. Funnel plot of the included studies.

© 2025 Aychiluhm SB et al. *JAMA Network Open*.
