# Supplementary material for: Interventions for Childhood Central Obesity: A Systematic Review and Meta-Analysis
Source: JAMA Netw Open. 2025 Apr 11;8(4):e254331. doi: 10.1001/jamanetworkopen.2025.4331 (PMC11992610; doi:10.1001/jamanetworkopen.2025.4331)
Supplement: Supplement 2. — Data Sharing Statement [file jamanetwopen-e254331-s002.pdf]

Data Sharing Statement

Aychiluhm. Interventions for Childhood Central Obesity. *JAMA Netw Open*. Published April 09, 2025. doi:10.1001/jamanetworkopen.2025.4331

Data
Data available: No
